# Aminopeptidase A contributes to biochemical, anatomical and cognitive defects in Alzheimer’s disease (AD) mouse model and is increased at early stage in sporadic AD brain

**DOI:** 10.1007/s00401-021-02308-0

**Published:** 2021-04-21

**Authors:** Audrey Valverde, Julie Dunys, Thomas Lorivel, Delphine Debayle, Anne-Sophie Gay, Sandra Lacas-Gervais, Bernard. P. Roques, Mounia Chami, Frédéric Checler

**Affiliations:** 1 INSERM, CNRS, IPMC, Team Labelled “Laboratory of Excellence (LABEX) DistAlz”, Institut de Pharmacologie Moléculaire et Cellulaire, Université Côte d’Azur, 660 route des Lucioles, Sophia-Antipolis, 06560 Valbonne, France; 2 CCMA-Université Côte d’Azur, Nice, France; 3 Faculté de Pharmacie, Université Paris-Descartes, 75006 Paris, France

**Keywords:** N-terminally-truncated Aβ, PE3-42Aβ, Aminopeptidase A, Inhibitors, ShRNA, Senile plaques, Dendritic spines, Behavior, Transgenic mice, Alzheimer Disease

## Abstract

One of the main components of senile plaques in Alzheimer’s disease (AD)-affected brain is the Aβ peptide species harboring a pyroglutamate at position three pE3-Aβ. Several studies indicated that pE3-Aβ is toxic, prone to aggregation and serves as a seed of Aβ aggregation. The cyclisation of the glutamate residue is produced by glutaminyl cyclase, the pharmacological and genetic reductions of which significantly alleviate AD-related anatomical lesions and cognitive defects in mice models. The cyclisation of the glutamate in position 3 requires prior removal of the Aβ N-terminal aspartyl residue to allow subsequent biotransformation. The enzyme responsible for this rate-limiting catalytic step and its relevance as a putative trigger of AD pathology remained yet to be established. Here, we identify aminopeptidase A as the main exopeptidase involved in the N-terminal truncation of Aβ and document its key contribution to AD-related anatomical and behavioral defects. First, we show by mass spectrometry that human recombinant aminopeptidase A (APA) truncates synthetic Aβ1-40 to yield Aβ2-40. We demonstrate that the pharmacological blockade of APA with its selective inhibitor RB150 restores the density of mature spines and significantly reduced filopodia-like processes in hippocampal organotypic slices cultures virally transduced with the Swedish mutated Aβ-precursor protein (βAPP). Pharmacological reduction of APA activity and lowering of its expression by shRNA affect pE3-42Aβ- and Aβ1-42-positive plaques and expressions in 3xTg-AD mice brains. Further, we show that both APA inhibitors and shRNA partly alleviate learning and memory deficits observed in 3xTg-AD mice. Importantly, we demonstrate that, concomitantly to the occurrence of pE3-42Aβ-positive plaques, APA activity is augmented at early Braak stages in sporadic AD brains. Overall, our data indicate that APA is a key enzyme involved in Aβ N-terminal truncation and suggest the potential benefit of targeting this proteolytic activity to interfere with AD pathology.

**Supplementary Information:**

The online version contains supplementary material available at 10.1007/s00401-021-02308-0.

## Introduction

Alzheimer’s disease (AD) is a devastating pathology that affects the aged population. At the histopathological level, AD is characterized by the accumulation of extracellular lesions, the senile plaques that are composed of Aβ40 and Aβ42 peptides and of intracellular neurofibrillary tangles that result mostly from hyperphosphorylation of Tau [8, 17, 56]. These two lesions are not exhaustive and among others, neuroinflammation, lysosomal/endosomal, mitochondrial dysfunctions as well as endoplasmic reticulum stress perturbations have been consistently observed in AD-affected brains.

The consistent failures of Aβ-directed clinical assays raised the possibility that the so-called amyloid cascade, that postulates amyloid-β (Aβ) as an etiological trigger of the pathology [25], could well be erroneous [26]. However, genetic evidences clearly indicate that “something linked to the Aβ-precursor protein (βAPP)” participates to AD pathological process. Hence, mutations responsible for early onset and aggressive forms of AD all affect either βAPP or the Aβ-generating enzyme (γ-secretase).

To reconcile indisputable genetic clues and recurrent failures of Aβ-directed clinical trials, one could envision additional APP-derived fragments distinct from Aβ, the contributions of which could have been underscored [39]. These include the β- and γ-secretases-derived APP C-terminal fragments C99 and C83, respectively [10], the recently described η-secretase generated fragments [3, 61] or AICD (APP Intracellular Domain) [36, 47]. In addition, several lines of evidence indicated that N-terminally truncated forms of Aβ correspond to the earliest and most abundant detectable Aβ species observed *post-mortem* in AD [53] as well as in down-syndrome-affected brains [27, 29]. These N-terminal fragments, that constitute a rather heterogeneous set of peptides [19], accumulate in the parenchyma as well as in the cerebrovascular wall of endothelial cells [30, 37].

One of the most abundant and toxic forms of these N-terminally truncated Aβ fragments is the pE3-42Aβ [18]. This fragment occurs early not only in compact but also diffuse plaques [30, 31]. Its importance has been well established by means of genetic and pharmacological approaches targeting glutaminyl cyclase (QC), the enzyme responsible for the cyclisation of glutamate in position three of Aβ [15, 55]. Thus, the depletion of the endogenous enzyme [34] or its pharmacological blockade by means of selective inhibitors [55] led to the abolishment of pE3-42Aβ production and significant improvement of cognitive functions in AD animal models. It should be emphasized that the cyclisation of the E3 residue could only occur after prior removal of the two first N-terminal amino-acids of Aβ. Therefore, the enzymes involved in these first exopeptidasic steps could be considered as rate-limiting enzymes conditioning the production of pE3-42Aβ and thereby, governing its toxic phenotypes.

Theoretical grounds suggested that APA, that display high catalytic avidity and affinity for acidic residues [9] could well be responsible for the removal of the aspartyl residue in position 1 of Aβ [57]. This hypothesis was indeed supported by an indirect pharmacological approach showing that APA specific inhibitors potentiated the recovery of intact full-length Aβ produced by human embryonic kidney cells (HEK293) overexpressing the Swedish mutated APP as well as in a cell-free system of Aβ production [57]. However, the direct evidence of the generation of Aβ2-X from Aβ was still lacking. Further, the putative consequences of APA modulation on AD-associated anatomical stigmata and cognitive defects remained to be established. Here, we definitely demonstrate that APA contributes to the removal of the first aspartyl residue of Aβ. Furthermore, we show that the pharmacological blockade or gene reduction of APA reduces this N-terminal truncation, shifts dendritic spine filopodia towards mature dendrites in ex vivo organotypic hippocampal slices cultures, reduces pE3-42Aβ- and Aβ-positive plaques and expressions and rescues memory defects observed in 3xTg-AD mice. Finally, we show that APA activity is augmented concomitantly to the early appearance of pE3-Aβ42 in sporadic AD human brains.

## Materials and methods

### Mass spectrometry

Synthetic Aβ40 from Bachem (3 µg) was incubated for 8 h with recombinant human APA (rAPA, 400 ng) (R&D System) with or without the APA inhibitor amastatin (100 µM) in a final volume of 200 μl of deionized water containing CaCl_2_ (1 mM). Samples were collected at different time periods (0, 2, 4, 6 and 8 h) then reactions were stopped after addition of 0.1% of formic acid and samples were subjected to spectroscopic analysis. Briefly, Aβ peptides were separated with Ultra-Performance Liquid Chromatography system (UPLC) (ThermoFisher) on a C18 column. Mass spectrometry data were acquired with a Q-Exactive *plus* mass spectrometer (ThermoFisher) operating in Full-Scan mode. Finally, Aβ fragments were identified using Xcalibur Quan-Browser software version 4.1.31.9.

### Cell culture, transfections and FACS selection

Mouse neuroblastoma N2a cells (ATCC, CCL131) were grown in DMEM supplemented with SVF (10%) and penicillin/streptomycin medium (10000U/ml, 1%), then transfected with lipofectamine 2000 (Invitrogen) according to manufacturer’s recommendations. Forty-eight hours after transfection, the selection was performed by addition of puromycin (1 μg/ml) and cells were maintained for 3 weeks in the same medium. For fluorescence-activated cells sorting (FACS), cells were scrapped with accutase solution (Sigma-Aldrich), centrifuged for 5 min at 600×g, then pellets were resuspended in phosphate-buffered saline (PBS), bovine serum albumin (BSA 0.5%), ethylene diamine tetra acetic acid (EDTA, 2.5 mM) buffer and filtered (0.45 µm). We used FACSAria III cell sorter (FSC/SSC parameter, BD Biosciences) to recover Green Fluorescent Protein (GFP)-positive and DAPI-labeled (DAPI 0.05ug/ml) cells and to discard dead cells.

### Viral production

Lentiviral particles were produced by co-transfecting two helper plasmids, delta8.9 (packaging vector) and VSV-G (envelope vector) and a transfer vector (pGFP-C-sh Lenti) into Lenti-X 293 T cell line (632,180; Clontech, Mountain View, CA, USA) as previously described [12]. Four shRNA constructs as well as a scramble control shRNA were inserted in the vector pGFP-C-sh Lenti (Cat. N°T R30023; Origene). Gene specific mouse shRNA sequence is: TL513554 ENPEP (Gene ID 13,809, Origen). We also produced Green, Green-APPwt and Green-APPswe lentiviruses (cloned in the lentiviral vector with an IRES- ZsGreen fluorescent tag (pHAGE-CMV-MCS-IRES-ZsGreen) [6], under the control of the CMV promoter. Viral titers were assessed using p24 ELISA (Cell Biolabs, San Diego, CA, USA).

### Organotypic hippocampal slices preparation and culture

Organotypic hippocampal slices preparations were achieved on C57bl6JRj mice (Janvier Labs, Le Genest Saint-Isle, France) 5 to 7 days after birth. Brains were quickly dissected out to retrieve hippocampi from both hemispheres, that were then sliced onto 400 µm sections and kept into slicing medium [Earles’ Balanced Salt solution (EBSS; 97.5%) and EBSS-HEPES (2.5%)]. Slices were transferred on sterile hydrophilic membrane millicell discs (Millipore, FHLC01300) placed in semiporous cell culture inserts (Millipore, 0.4 µm) containing culture medium (Minimum Essential Medium Eagle (MEM) + Glutamax-1 (50%), EBSS (18%), EBSS (13%)/D-glucose (5%), penicillin–streptomycin 5000U/ml (1%), Horse serum (25%) and Nystatin 10000U/ml (0.06%). Slices were infected 2 h after plating with 2 µl of lentiviruses encoding for lenti-Green (virus titer: 2.44 × 10^10^), lenti-Green-APPwt, (virus titer: 2.21 × 10^10^) or lenti-Green-APPswe, (virus titer: 2.21 × 10^10^). Slices were kept at 37 °C, 5% CO_2_ for 9 days before treatments and imaging experiments.

### Human brain samples and preparation for APA activity

Human brain samples were obtained thanks to the “NeuroCeb” Brain Bank run by a consortium of associations: CSC (Cerebellar ataxias), ARSEP (association for research on multiple sclerosis) and France Parkinson. All procedures with human brain samples were performed in accordance with the ethical standards of both institutional and national research committees as well as the 1964 Helsinki declaration and amendments. Individual consents were signed by the patients or their close relatives in their name and in accordance with the French Bioethical Agreement (AC-2013–1887). Cases were anonymized and information regarding age, sex and neuropathology (Braak and Thal stages, amyloid angiopathy) are provided in Suppl. Table 1, online resource. Three ALS cases were included as controls since they did not display NFT according to their Braak stage, were devoid of Aβ as underlined by their Thal stage and were free of amyloid angiopathy (see Suppl. Table 1, online resource). Further, we established that these cases display APP, Aβ, APP C-terminal fragments (CTFs) and neurofilament expressions similar (not statistically significantly different from other controls, (data not shown)). Braak stages I–III samples display tau-related pathology and no amyloid pathology and, as such, could also be considered as “controls with AD-related pathology”. Tissue lysates from human temporal cortices were obtained after mechanical homogenization (in Tris 10 mM) with a Teflon-glass potter followed by a breve sonication. APA enzymatic activity was determined as described below.

### APA activity measurements

Hippocampi or FACS-sorted cells were homogenized in Tris buffer (10 mM, p7.5). APA activity (50 µg of homogenates) was measured in assay buffer (Tris 50 mM, pH 7.5 containing CaCl_2_ 1 mM) [57] with or without the APA inhibitor RB150 (100 μM) by means of Glu‐7-amino-4-methylcoumarin (Glu-7-AMC, 100 μM, Santa Cruz Biotech) as substrate. Initial velocity recordings of fluorescence were performed at 360 nm and 460 nm excitation and emission wavelengths, respectively, as described [9].

### Preparation of insoluble fractions and Aβ quantitation by Elisa

Human brain samples and dissected hippocampi from wild-type and 3xTgAD mice were homogenized in radioimmunoprecipitation assay buffer (RIPA) (Tris 50 mM; pH 7.4 containing NaCl (150 mM), EDTA (1 mM), Triton X100 (1%), deoxycholate (0.5%), sodium dodecyl sulfate (SDS, 0,1%) and complete protease inhibitor mixture Sigma) as previously described [48]. After homogenization with a teflon-glass potter, homogenates were centrifuged (100,000 g, 1 h, 4 °C), and supernatants were kept as soluble fractions. Pellets containing insoluble material were resuspended in formic acid (70%), centrifuged (100,000 g, 1 h, 4 °C), and then supernatants were neutralized to pH 7.5 with Tris–HCl (1 M, pH 10.8) containing betaine (25 mM) (at a 1/25 dilution) and referred to as the insoluble fractions. For pE3-40/42Aβ detection, insoluble fractions were concentrated by speedvac at 40 °C before neutralization with Tris–HCl–betaine.

Human Aβ40, Aβ42 and pE3-xAβ peptides levels were measured in the aforementioned soluble and insoluble fractions using sandwich enzyme-linked immunosorbent assay kits (BioSource [Invitrogen], France and IBL international, respectively) as already described [38].

### SDS/PAGE and western blot analyses

N2a cells were scrapped, mechanically lyzed in homogeneization buffer HEPES (5 mM) containing sucrose (250 mM) and EDTA (1 mM) then centrifuged for 15 min at 800 × g. Supernatants were collected and centrifuged for 1 h at 20,000 × g then pellets were resuspended in RIPA buffer with a protease inhibitor cocktail. 50 μg of proteins (membranous or soluble fractions) was analyzed on Tris–glycine 10% acrylamide gels, then wet-transferred onto nitrocellulose. Membranes were incubated overnight with antibodies against β-actin (A5316-sigma, mouse), Glyceraldehyde-3-phosphate dehydrogenase (GAPDH; MAB374-sigma, mouse) or APA (ab36122, goat) and immunoreactivities were revealed as previously described [24].

### DAB and Immunofluorescence staining

shScr- or shAPA-infected 3xTg-AD mice were anesthetized by intraperitoneal injection of ketamine (120 mg/kg) and xylazine (24 mg/kg) and intracardially perfused with PBS followed by paraformaldehyde (4%). Brains were collected and embedded in paraffin then sliced (8 µm of thickness) with a microtome apparatus. Mice or human brain slices were treated with formic acid or heat treatment in citrate buffer during 30 min and then incubated overnight at 4 °C with anti Aβ42 antibody (d:1/1000, Invitrogen), anti-82E1 (d:1/1000, mouse, IBL International), anti-APA (d:1/500, goat, ab36122) or anti pE3-xAβ (N3pE antibody, d:1/50, rabbit, IBL International). After three washes, sections were incubated for one hour with adequate secondary horseradish peroxidase antibodies (Interchim, Montluçon, France) or biotynilated anti-rabbit antibody (Vector laboratories, Burlingame, California, USA) for Aβ42, APA and pE3-xAβ, respectively. For pE3-xAβ and APA detection (Suppl. Figure 10, online resource), signal amplification was achieved with the Vectastain Elite ABC-HRP kit. Slices were then revealed with the DAB-ImmPACT system (Vector Laboratories) for Aβ42, APA and pE3-xAβ. Plaques were quantified from four hippocampal slices of 3xTg-AD (shScr, *n* = 5) or (shAPA, *n* = 8) mice. For co-localisation experiments, slices were immuno-stained with fluorescent antibodies, Alexa-488- or Alexa-594-conjugated (Molecular Probe, d:1/1000). Nuclei were stained with Dapi (Molecular Probes, d:1/20,000). Pictures were taken with a confocal Leica TCS SP5 microscope (for Immunofluorescence) and with DMD108 Leica microsystem (for DAB staining).

### Cells imaging

N2a cells were grown in 6-well plates at a density of 100 000 cells/well. Five days after infection, cells were fixed for 20 min with paraformaldehyde (PFA, 4%), then washed three times with PBS, permeabilized with Triton (0.1%) for 5 min and blocked for one hour with BSA (5%)/Tween (0.05%). Primary antibodies against APA were added overnight (1/500 dilution). Cells were then rinsed three times with PBS then incubated for one hour with secondary anti-Goat antibody (Interchim). Nuclei were stained with Dapi (1/20,000 dilution). Cells were then rinsed again with phosphate-buffered saline (PBS, 1X). Slices were mounted and cover-slipped with the Vectamount medium (Vector Laboratories) before imaging with the Biotek Cytation 5 microscope. Six fields per well were automatically acquired (brightfield and fluorescent images) using the acquisitions parameters (× 20 magnification, numeric aperture 0.45, and adapted excitation and emissions filters: DAPI (ex 377; em 447); GFP (ex 469; em 525); Txs Red (ex 586; em 647)). We used a macro in Image J software to quantify the red fluorescence intensity in GFP-positive cells.

### Imaging of spine morphology and quantitation

Slices were left attached on the millicell membranes and mounted with mounting solution, cover-slipped and dried before imaging. Spine morphology was assessed with LSM 780 microscope, Plan-Apochromat 63 × /1.40 Oil DIC M27 lens, zoom 3.0 and pinhole at 52 µm on ten to twenty different dendrite segments (obtained from two independent experiments). The quantitation of spine structures (mature, stubby and filopodia) was performed manually as previously described [51, 52] using “false” colors images ZEN software.

### Animals

3xTgAD mice [46] and wild-type (WT; non-transgenic) mice were maintained from breeding pairs provided by Dr. LaFerla (Irvine, USA). Animals were housed with a 12:12 h light/dark cycle and were given free access to food and water. All experimental procedures were in accordance with the European Communities Council Directive of 22 September 2010 (2010/63/EU) and approved by the French Ministry of Higher Education and Research (Project number APAFIS#9645–2,017,012,315,473,838) and by Côte d’Azur University Animal Care and Use Committee.

### In vivo pharmacological treatments

A specific and potent inhibitor of APA, RB150 [20] was administered daily during six weeks by intraperitoneal injections at a dose of 15 mg/kg/day before animal sacrifices and analyses.

### Viral stereotaxic injections

Two- to three-month-old WT and 3xTg-AD mice were anesthetized by intraperitoneal injection with a mixture of ketamine (100 mg/kg) and xylazine (10 mg/kg). Mice were placed in a mouse head holder and lentiviral vectors were stereotactically injected bilaterally into the subiculum region (3 μl) with viral particles (1 × 10^10^–2 × 10^10^ viral particles) at the following coordinates Sub: a/p: ± 3.8, m/l ± 2.5, d/v, − 2.0 (The Mouse Brain in stereotaxic coordinates, Second Edition, Elsevier Academic Press).

### Barnes maze

Spatial learning and memory of mice were studied using a dry land-based rodent procedure [41]. First, mice performed three trials during four days on a spatial acquisition protocol allowing animals to retrieve a box placed under a hole of the Barnes Maze apparatus. Then, mice were assessed for memory with a probe trial consisting in the removal of the base and 60 s allotted time for mice to find the target place. Several parameters were evaluated: latency to reach the target site, number of quadrant crossing, latency and distance in the quadrant of the target site. Data were analyzed with the Animaze 6.1 Software.

### Morris water maze

Morris Water Maze (MWM) task was assessed in a circular 90 cm pool filled with a white opaque solution. Pool was placed in a room surrounded by visual clues. Maze was performed as previously extensively described [5, 12]. Data were analyzed with the Animaze 6.1 Software.

### Rotarod performance test

Motor performance of mice was measured on a rotarod apparatus (Bioseb, model LE8200) as previously described [5]. Briefly, mice were placed on a rotating rod accelerating from 4 to 40 rpm. Latency to fall was then tested for all animals. Three trials were performed during a period of 5 min for each animal.

### Open field test

Mice anxiety and exploratory behavior were recorded by an Open Field test. Briefly, time spent in the center of the box (40 cm × 40 cm) was recorded during 10 min per animal.

### Statistical analysis

Organotypic hippocampal slices (spine morphology differences after treatment with various synthetic Aβ (40, 42 or pE3-42) peptides, or comparison of RB150-treated APPswe or APPwt-infected slices) and mass spectrometry (Aβ2-40/Aβ40 ratios) were analyzed by One-way ANOVA, Kruskal–Wallis.

For behavior experiments with WT and 3xTg-AD mice injected with shRNA lentiviruses, all statistical analyses were realized using InVivoStat software. For each statistical analysis, normal distribution of residuals and homogeneity of variance were analyzed by checking normal probability plot and residual versus predicted plot. For Barnes Maze and MWM task, escape latency was analyzed by mixed model ANOVA with repeated measure (genotype × injection × training day) and followed by Benjamini–Hochberg correction. Concerning statistics of probe-phase MWM and Barnes Maze, open-field and rotarod, the influences of both injection and genotype were considered by 2-way ANOVAs (genotype × injection) with multiple comparisons adjusted by Benjamini–Hochberg method. For open-field statistical analyses, data were log-10-transformed to respect homoscedasticity assumptions. For immunohistochemistry and ELISA, we used the Mann–Whitney test. The number, area and perimeter of plaques were analyzed by automatic-macro program on Image J software and every point corresponds to 5 pictures for shScr-3xTg-AD (*n* = 5) or shAPA 3xTg-AD (*n* = 8) mice.

## Results

### APA-mediated generation of Aβ 2–40 in vitro

**Fig. 1:**
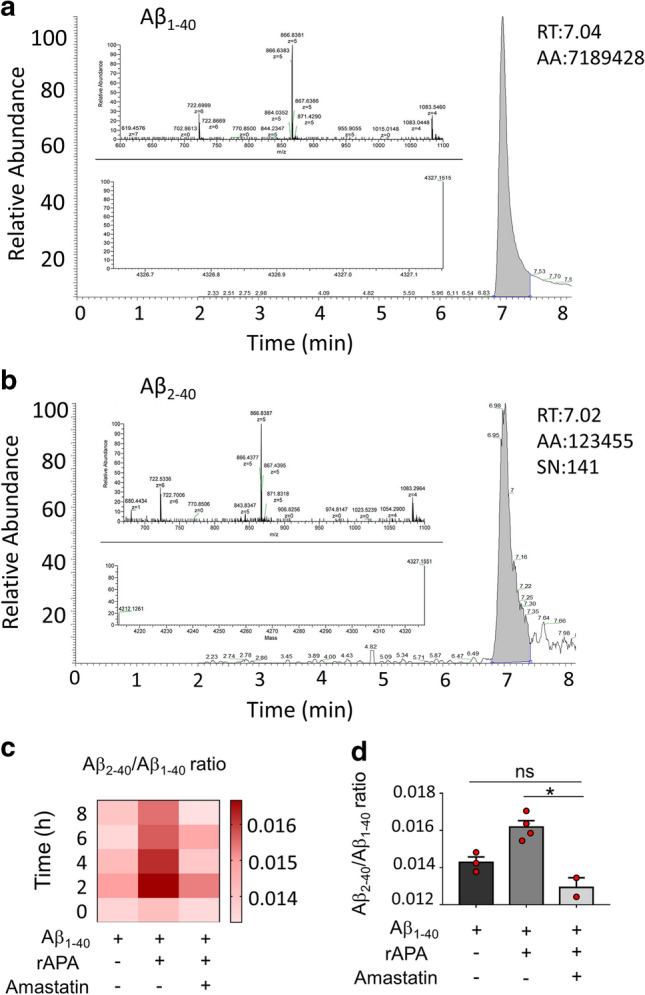
APA catalyzes the production of Aβ2-40 from Aβ1-40. **a, b** HPLC analyses of Aβ1-40 (**a**) and Aβ2-40 (**b**) peptides after incubation of Aβ for 6 h with recombinant APA, as described in the methods, with their spectrum and their spectral deconvolution revealing m/z values (4327.1515 and 4212.1261, respectively). RT: retention time, AA: Average Area, SN: Signal Noise. **c** Aβ2-40/Aβ1-40 ratio is represented over indicated time in the presence or absence of human recombinant APA (rAPA), with or without amastatin as described in Methods. **d** Quantitation of Aβ2-40/Aβ1-40 ratio at 4 h of incubation in conditions identical to those in panel **c**. Statistical analyses were performed using one-way ANOVA with Dunnett’s multiple comparisons post-test obtained in three independent experiments. *p < 0.05

We previously showed in vitro, that the incubation of human recombinant APA (rAPA) with synthetic Aβ reduced full-length Aβ recovery in an APA-inhibitor sensitive manner [57], but the exact nature of the cleavage mediated by APA was not established. According to Aβ sequence and APA specificity [9], it was expected that APA would trigger the removal of the first N-terminal aspartyl residue. This theoretical assumption was verified by mass spectrometry analysis. Thus, we found that rAPA hydrolyses Aβ1-40 synthetic peptide that is converted into a smaller fragment unambiguously identified by its mass as Aβ2-40 (Fig. 1a, b). Of note, the calculation of Aβ2-40/ Aβ1-40 ratios (Fig. 1c) indicates that it increases transiently, suggesting that either recombinant APA contains a few contaminating exopeptidases or, alternatively, that APA itself could display a wide specificity explaining an atypical secondary cleavage of Aβ2-40. However, we show that Aβ2-40 peptide formation was abolished in the presence of the acidic protease inhibitor amastatin known to act as a potent APA inhibitor (Fig. 1c, d) [2]. This set of data confirms that APA displays the ability to truncate Aβ1-40 at its N-terminus, thereby yielding Aβ2-40.

### APA-mediated alteration of synaptic maturation

**Fig. 2:**
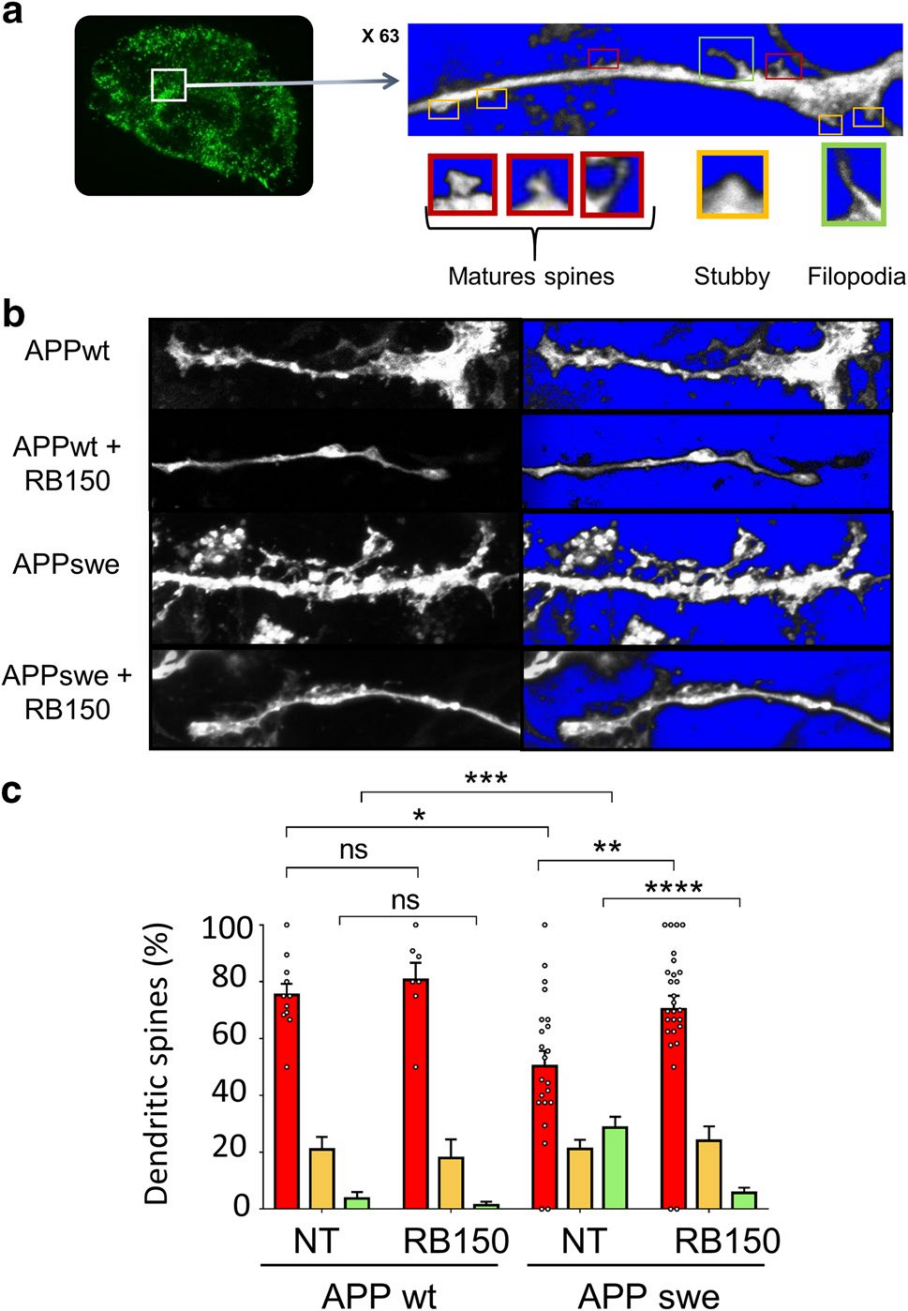
Pharmacological blockade of APA influences dendritic spines morphology. **a** Typical illustration of hippocampal organotypic slices infected with Green lentivirus. Red boxes highlight mature spines, yellow box shows stubby spines and green box shows filopodia. **b** Representative images of dendritic morphology in hippocampal organotypic slices infected with either APPwt or APPswe lentiviruses in absence or in the presence of RB150 as described in Methods. **c** Quantification of spines types expressed as percent of total spines (NT, non treated). Statistical analyses were performed using Kruskal–Wallis with Dunnett’s multiple comparisons post-test (APPWT NT: *n* = 11; APPWT RB150: *n* = 7; APPSwe NT: *n* = 22; APPSwe RB150: *n* = 27, *p < 0.05; **p < 0.005; ***p < 0.0005; ****p < 0.0001). ns, not statistically significant

**Fig. 3:**
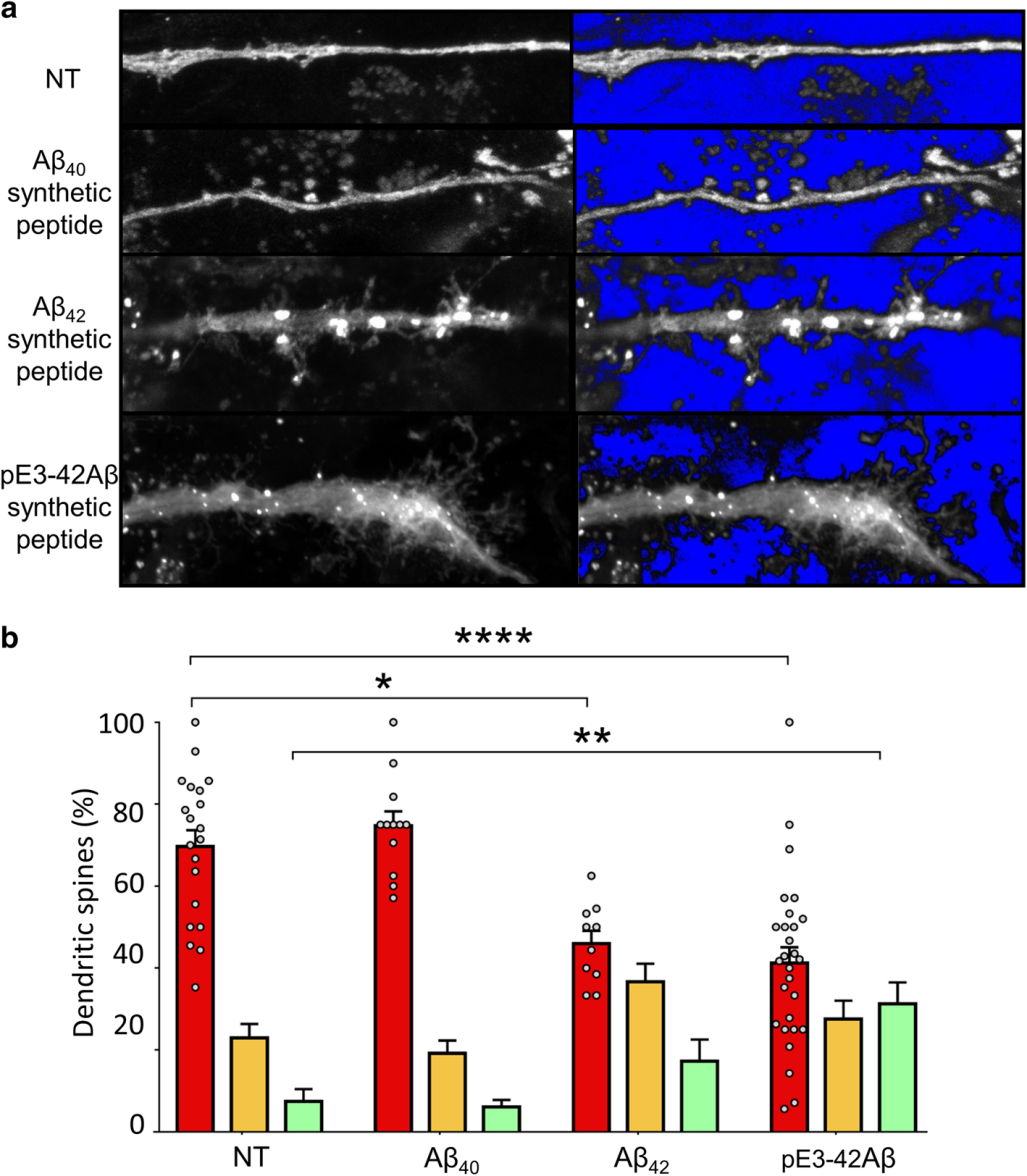
Aβ42 and pE3-42Aβ influence dendritic spines morphology. **a** Slices were infected with Green-expressing lentiviruses then exposed overnight to synthetic Aβ40, Aβ42 or pE3-42Aβ (150 ng). **b** Quantification of spines types expressed as percent of total spines (NT, non treated). Statistical analyses were performed using a One-way Anova, Kruskal–Wallis test (Green NT: *n* = 20; Green Aβ40: *n* = 12; Green Aβ42: *n* = 10; Green pE3-42Aβ: *n* = 28, where. *p < 0.05; **p < 0.005; ****p < 0.0001). *n* represents the number of dendrites obtained in three organotypic slices from two independent experiments

We examined the functional consequences of targeting APA on synaptic plasticity, through the analyses of the morphology of dendritic spines. To address this question, we took advantage of hippocampal organotypic slices prepared from young mice (5 to 7 days after birth) that were infected with lentiviruses expressing either wild-type APP (APPwt) or APP bearing the Swedish mutation (APPswe) expressed together with Green protein with IRES system (see Methods). Spine imaging allowed us to categorize three spine morphotypes reflecting their degree of maturity, i.e. mature spines, stubby spines or filopodia (Fig. 2a). Comparison of APPwt and APPswe hippocampal organotypic slices revealed a reduction of the number of mature spines concomitant to a significant increase in the number of immature filopodia in APPswe-infected slices (Fig. 2b, c and Suppl. Figure 1, online resource). Of utmost interest, the increased number of filopodia observed in APPswe slices was fully rescued by RB150, a specific and selective APA inhibitor [20] (Fig. 2b, c), thus indicating that an APA-dependent catalysis accounted for synaptic maturation defects. Since the APPswe increases Aβ production [7, 13], this could indicate that either Aβ itself or one of its derived catabolites could alter synaptic plasticity in this model. This was verified on hippocampal slices derived from mice infected with a lentivirus encoding for the green fluorescent protein only that were incubated with synthetic Aβ1-40, Aβ1-42 or pE3-42Aβ peptides (Fig. 3). Interestingly, we observed that Aβ1-40 did not affect spine morphology while both Aβ1-42 and pE3-42Aβ lowered the percentage of mature spines and concomitantly increased stubby and filopodia spines (Fig. 3a, b). It should be noted that the extent of percentage of filopodia was higher in pE3-42Aβ- than in Aβ1-42-treated slices (Fig. 3b). Altogether, these data indicated an APPswe-linked toxic effect on dendritic spines that can be reversed by APA inhibitors and mimicked by pE3-42Aβ peptide, hence suggesting that APA could contribute to the synaptic morphology alterations observed in AD.

### shRNA APA reduces the recovery of pE3-42Aβ and lowers the number of pE3-42Aβ- and Aβ-positive plaques in 3xTg-AD mice

**Fig. 4:**
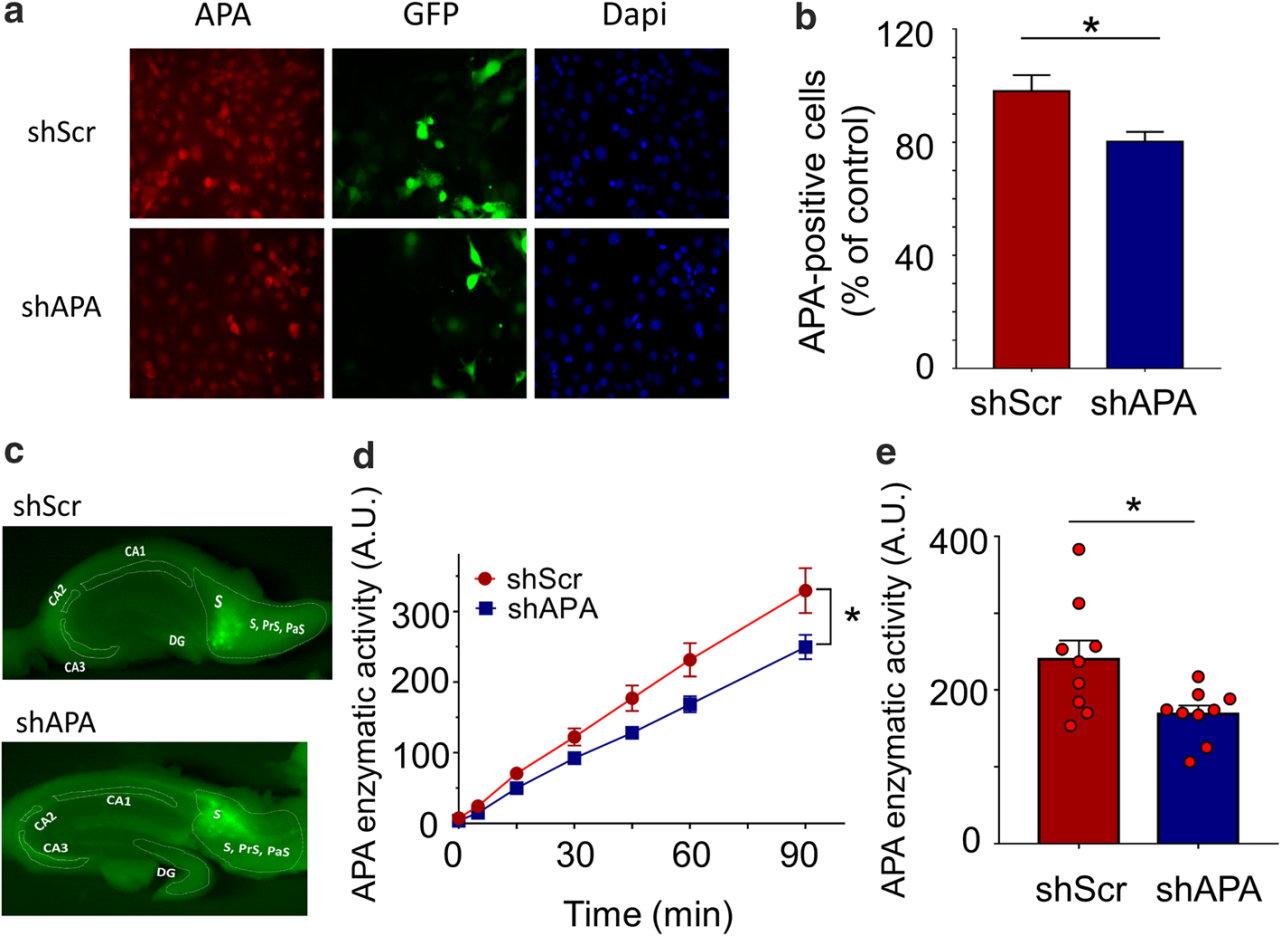
shRNA-dependent modulation of endogenous APA expression and activity in N2a cells and in infected mouse brain. **a** Representative images showing N2a cells transduced with lentiviruses encoding either shRNA targeting APA (shAPA) or scramble shRNA (shScr, control). Images are representatives of immunofluorescences for APA (red), GFP (green) and Dapi (blue) stainings. **b** Graph represents quantitation of APA-positive N2a cells obtained using cytation imaging. Analyzed cells *n* = 520 for shScr and *n* = 539 for shAPA obtained in 4 independent determinations; *p < 0.05 (Mann–Whitney statistical test). **c** Representative mouse brain slices showing shRNA distribution (GFP-positive cells) one month after stereotaxic injection of indicated lentiviruses. **d** Kinetic analysis of RB150-sensitive APA enzymatic activity fluorimetrically measured in hippocampi of infected mice as described in methods. **e** Illustration of total and RB150-sensitive Glu-7AMC hydrolyzing activity at 60 min. (*n* = 9, statistical analysis: Wilcoxon test in D and Mann–Whitney in E. *p < 0.05

To examine the influence of endogenous APA on AD plaques, we first designed and validated APA-directed short hairpin ribonucleic acids (shRNA). Four sequences were delineated in silico (Suppl. Figure 2a, online resource) and examined for their ability to down-regulate cellular expression and activity of APA. All, but not APA-B, constructs allow reduction of APA expression (Suppl. Figure 2 b, c) online resource) and RB150-sensitive activity (Suppl. Figure 2d, online resource). We selected shRNA APA-A, which decreases APA expression and enzymatic activity by about 30% (Suppl. Figure 2c, d, online resource). Then, shRNA APA-A or control scramble shRNA (thereafter referred to as shAPA or shScr) was subcloned in lentivirus vectors co-expressing GFP and produced (titers were 1.38 × 10^10^ and 1.71 × 10^10^, respectively). Their transduction in neuroblastoma N2a cells shows a statistically significant reduction by about 20% of APA expression (Fig. 4 a, b). For in vivo assessments, we chose to use the 3xTg-AD mice that recapitulate most of AD-related lesions [46] and have been used consistently as a relevant AD mouse model. The certainty of the stereotaxic delivery of shAPA and shScr in 3xTg-AD mice brain subiculum, a brain zone altered in AD [33], was ascertained by examining green fluorescence that was indeed observed in the subiculum region (Fig. 4c). Importantly, shAPA-infected mice display about 20–30% reduction of APA activity as compared to shScr-infected mice (Fig. 4c).

**Fig. 5:**
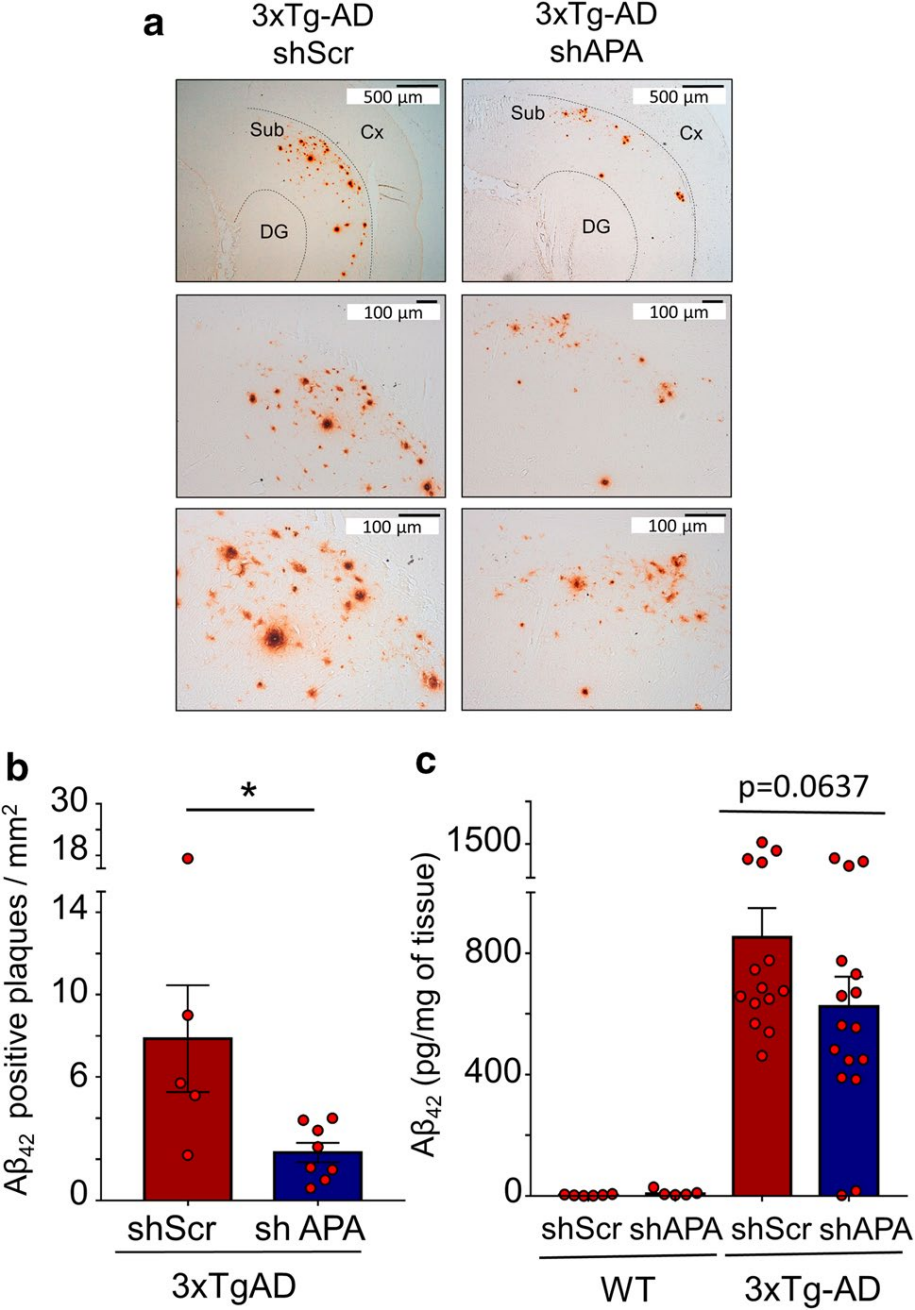
Genetic down-regulation of endogenous APA reduces Aβ42-positive plaques and Aβ42 expression in 3xTgAD mice brain. **a** Immunohistochemical analysis of Aβ42-like immunoreactivity in twelve-month-old 3xTgAD transgenic mice injected with shScr or shAPA. Scale bars = 100 µm or 500 µm. **Cx, cortex; DG, dentate gyrus; Sub, subiculum. b** Graph represents the number of Aβ1-42-positive plaques per mm^2^. Data represent means + / − SEM of 5 (shScr) and 8 (shAPA) mice (4 slices per mouse). Statistical analysis was performed using Mann–Whitney test. **c** Aβ42 levels detected by ELISA in insoluble fractions prepared from 12-month-old 3xTgAD transgenic mice injected with shAPA (*n* = 16) or shScr (*n* = 14). Data are expressed in pg of Aβ42 per mg of tissue. *p < 0.05

**Fig. 6:**
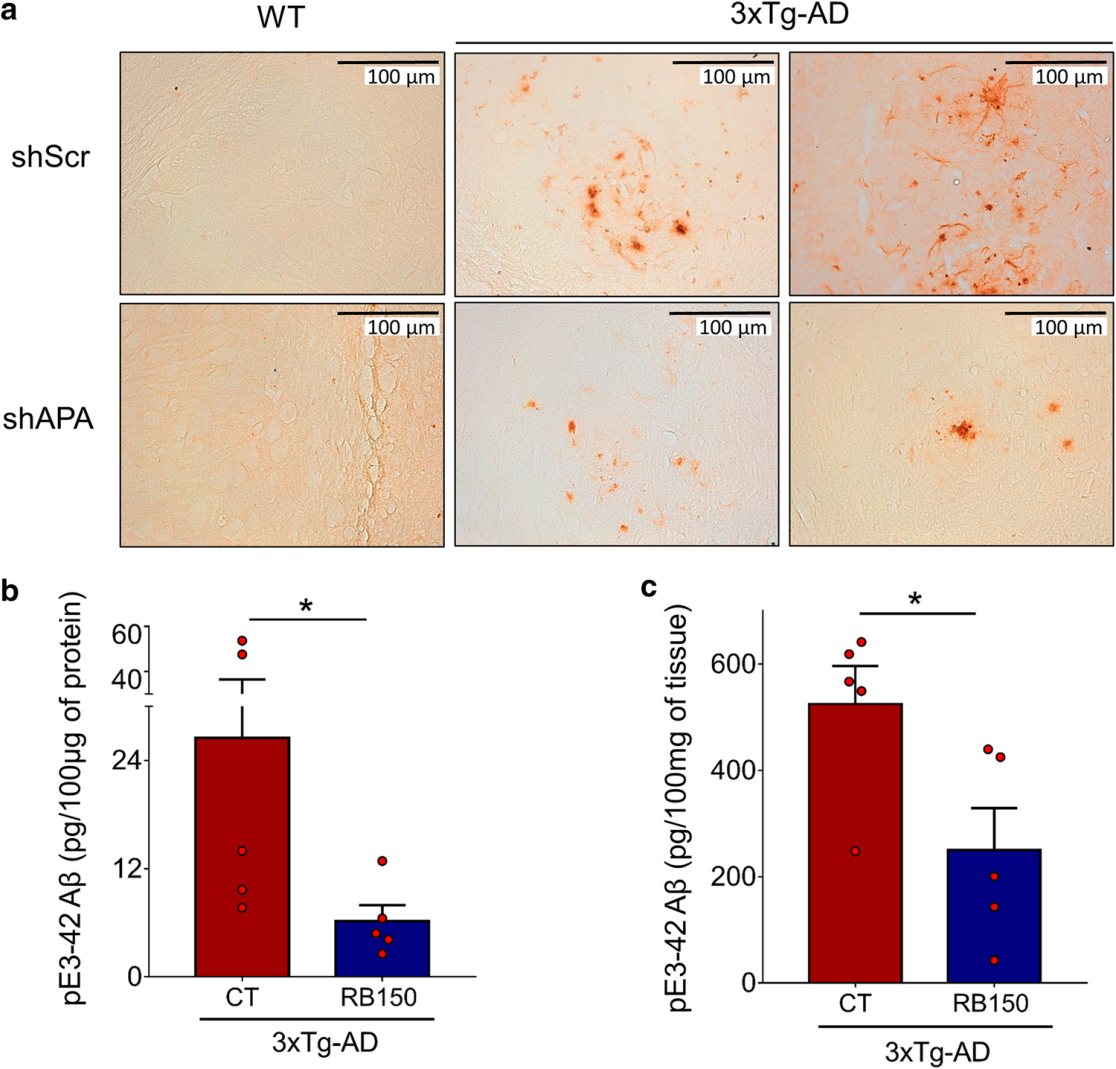
Genetic and pharmacological modulations of APA reduce pE3-xAβ-positive plaques and pE3-42Aβ level in 3xTg-AD hippocampi. **a** Immunohistochemical analysis of pE3-xAβ in 12-month-old 3xTgAD transgenic mice injected with shScr or shAPA. Quantification of pE3-42Aβ levels in soluble (**b**) and insoluble (**c**) fractions prepared from hippocampi of 15-month- old WT or 3xTgAD mice treated daily 6 weeks by oral gavage with either serum (control, CT) or RB150 (WT CT: *n* = 4; WT + RB150: *n* = 5; 3xTgAD CT: *n* = 5; 3xTgAD + RB150: *n* = 5). Statistical analysis was performed using Mann–Whitney test. *p < 0.05

We then examined whether APA reduction could influence the density and morphology of Aβ plaques in 12-month-old 3xTg-AD mice brain. We observed a drastic reduction of the density of Aβ42-positive plaques (Fig. 5a, b and Suppl. Figure 3, online resource) but not in the mean plaques perimeter or areas (Suppl. Figure 4 online resource). This was accompanied by a slight reduction of insoluble Aβ42 (Fig. 5c), an Aβ species thought to reflect its aggregated form seeded in plaques. Of interest, RB150 also drastically reduced the number of Aβ42-positive plaques (Suppl. Figure 5, online resource). We also examined the presence of pE3-Aβ-positive plaques and their susceptibility to APA gene reduction. At 12 months of age, pE3-Aβ-positive plaques were not detected in shScr- and shAPA-treated WT mice (Fig. 6a, left panels) and as expected, readily detectable in 3xTg-AD mice (Fig. 6a, middle and right upper panels). Interestingly, shAPA administration reduces the density of pE3-42Aβpositive plaques (Fig. 6a, compare middle and right panels). We also examined the effect of the pharmacological blockade of APA at 15-month-old mice. Interestingly, in agreement with pE3-42Aβ-positive plaques detection, both soluble (Fig. 6b) and insoluble (Fig. 6c) levels of pE3-42Aβ were high in 3xTg-AD and virtually undetectable in shScr and shAPA-WT mice (data not shown). Of importance, pE3-42Aβ loads were significantly reduced upon chronic treatment with RB150 (oral gavage administration for 6 weeks) in 3xTg-AD mice (Fig. 6b, c). Overall, our data demonstrate that APA-mediated production of pE3-42Aβ directly influences both Aβ1-42 and pE3-42Aβ-positive plaques and recoveries in 3xTg-AD mice. Of note, we showed that indeed, there existed co-localization between APA and Aβ in the subiculum indicating an enzyme/substrate topological co-existence, a prerequisite for envisioning functional APA-mediated production of pE3-Aβ (Suppl. Figure 6, online resource).

Besides Aβ lesions, AD brains have been shown to display lysosomal morphology alterations. Thus, we examined the putative influence of APA gene reduction on lysosomal morphology by electron microscopy and did not unravel modifications (data not shown). Further, as autophagy is an intracellular degradation machinery directly linked to lysosomes [63], we examined the induction of basal autophagy through the expression level of the autophagy substrate SQSTM1/p62 and the conversion of soluble microtubule-associated protein 1A/1B-light chain 3 (LC3-I) to lipid bound LC3-II and quantified LC3II/LC3I ratio allowing the quantification of autophagic flux [35]. Our data indicate that although, as described [60], LC3I and LC3II appeared to be enhanced in 3xTg-AD vs Wt mice, shAPA did not modulate LC3 expressions in 12-month-old 3xTg-AD mice brain (data not shown). In addition, p62 was not affected by APA reduction (data not shown). Overall, our data indicate that APA does not control lysosomal physiology and autophagic process.

### Pharmacological blockade and gene reduction of APA alleviate learning and memory defects in 3xTg-AD mice

**Fig. 7:**
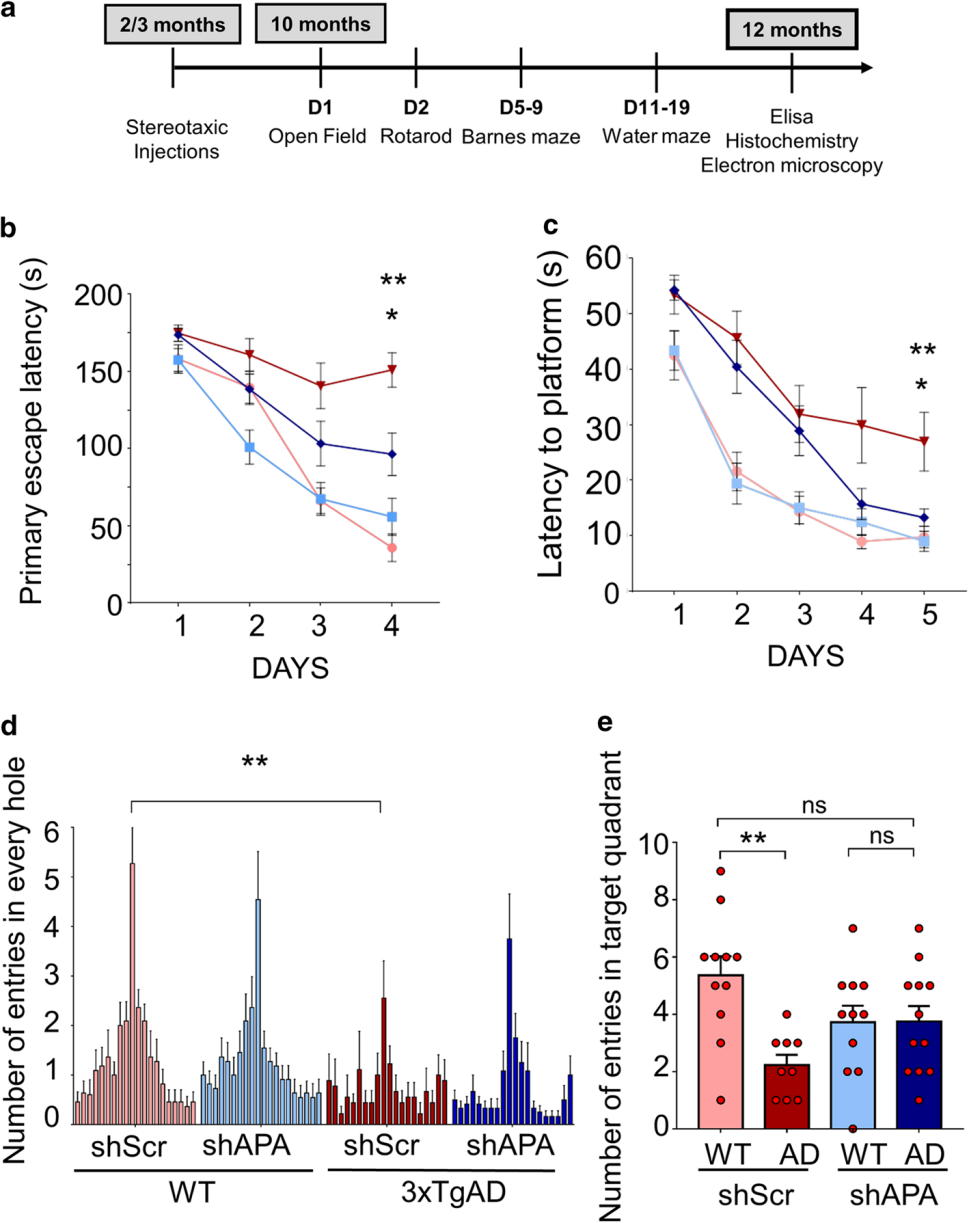
Genetic down-regulation of APA influences learning and memory in 3xTgAD mice model. **a** Time schedule of analyses of mice subjected to stereotaxic injections. **b**, **c** Graphs represent the primary escape latency to find the target hole in the Barnes maze (**b**) and the latency to reach the platform in the Morris Water Maze (**c**) for WT and 3xTgAD mice infected with shScr (light red and dark red, respectively) or shAPA (light blue and dark blue, respectively). **d**, **e** Show the number of entries in every hole of the Barnes maze (**d)** and the number of entries in target quadrant of the MWM (**e**) in mice and treatments described above. Statistical analyses were performed using InVivoStat (WT shScr: *n* = 11; WT shAPA: *n* = 11; 3xTgAD shScr: *n* = 9; 3xTgAD shAPA: *n* = 12) by 2-way ANOVA. Repeated measures parametric analysis: genotype x shRNA x day: *p* < 0.05 for both MWM and Barnes Maze. Post-hoc comparisons: 3xTg-AD shScr vs 3xTg-AD shAPA, *p < 0.05; 3xTg-AD shScr vs. WT shScr, **p < 0.005. Probe test: genotype × shRNA, *p < 0.05. Post-hoc comparisons: **p < 0.005. Genotype difference between 3xTgAD and WT for number of entries in target hole: **p < 0.005). *ns* not statistically significant

The 3xTgAD mouse model not only recapitulates most of anatomical hallmarks observed in AD pathology but also displays cognitive alterations in memory and learning tasks [58] that are characteristic defects observed in AD patients [32]. We chose the Morris Water Maze (MWM) and Barnes Maze, two well-known spatial learning tests, as readout of learning tasks. Of note, the Barnes Maze was documented as the most sensitive test for detecting spatial memory defects as early as at 6,5 month-old in 3xTg-AD mice [59]. We first examined the influence of shScr- ten months after stereotaxical administration in the subiculum of Wt and 3xTg-AD mice (see procedure timeline in Fig. 7a). As expected, in the Barnes Maze, average primary escape latencies (i.e., the time to identify the target hole the first time in each day) were significantly longer in shScr-3xTg-AD than in shScr-Wt mice (Fig. 7b) while the total number of entries in every hole was lower in the former mice (Fig. 7d). Further, we observed an enhanced latency to reach the platform in the Morris water maze at day 5 (Fig. 7c) and a drastically reduced number of entries in the target quadrant (Fig. 7e) in shScr-3xTg-AD mice.

We then examined the influence of APA reduction ten months after shAPA stereotaxical administration in the subiculum of mice (see procedure timeline in Fig. 7a). Interestingly, shAPA administration in 3xTg-AD mice significantly reduced the primary escape latency at day 4 (Fig. 7b), and fully rescued the latency to the platform (Fig. 7c). Our data also indicate that shAPA modified the distribution of the number of entries in 3xTg-AD mice, illustrated by a less randomness and more focused distribution of entries in the target hole (Fig. 7d and suppl. Figure 7a, online resource) and fully rescued the alteration observed in the number of entries in the target quadrant in shScr-3xTg-AD mice (Fig. 7e). Of note, the number of entries to target (NW) zone was significantly reduces in shScr-3xTg-AD vs shScr-Wt mice and returned to control upon shAPA injection in 3xTg-AD mice (Suppl. Figure 7b, online resource). Of importance, although, as was previously established [23], we observed a better motor coordination in shScr-3xTg-AD mice than in shScr-WT mice (Compare light and dark red bars in Suppl. Figure 8, online resource), the above-described shAPA-related behavioral modifications were not due to shAPA-linked alterations of motricity (Suppl. Figure 8a, online resource), anxiety (Suppl. Figure 8a, online resource) or visual acuity (Suppl. Figure 8c, online resource) as verified using rotarod, open field tests and visual cued test, respectively.

**Fig. 8:**
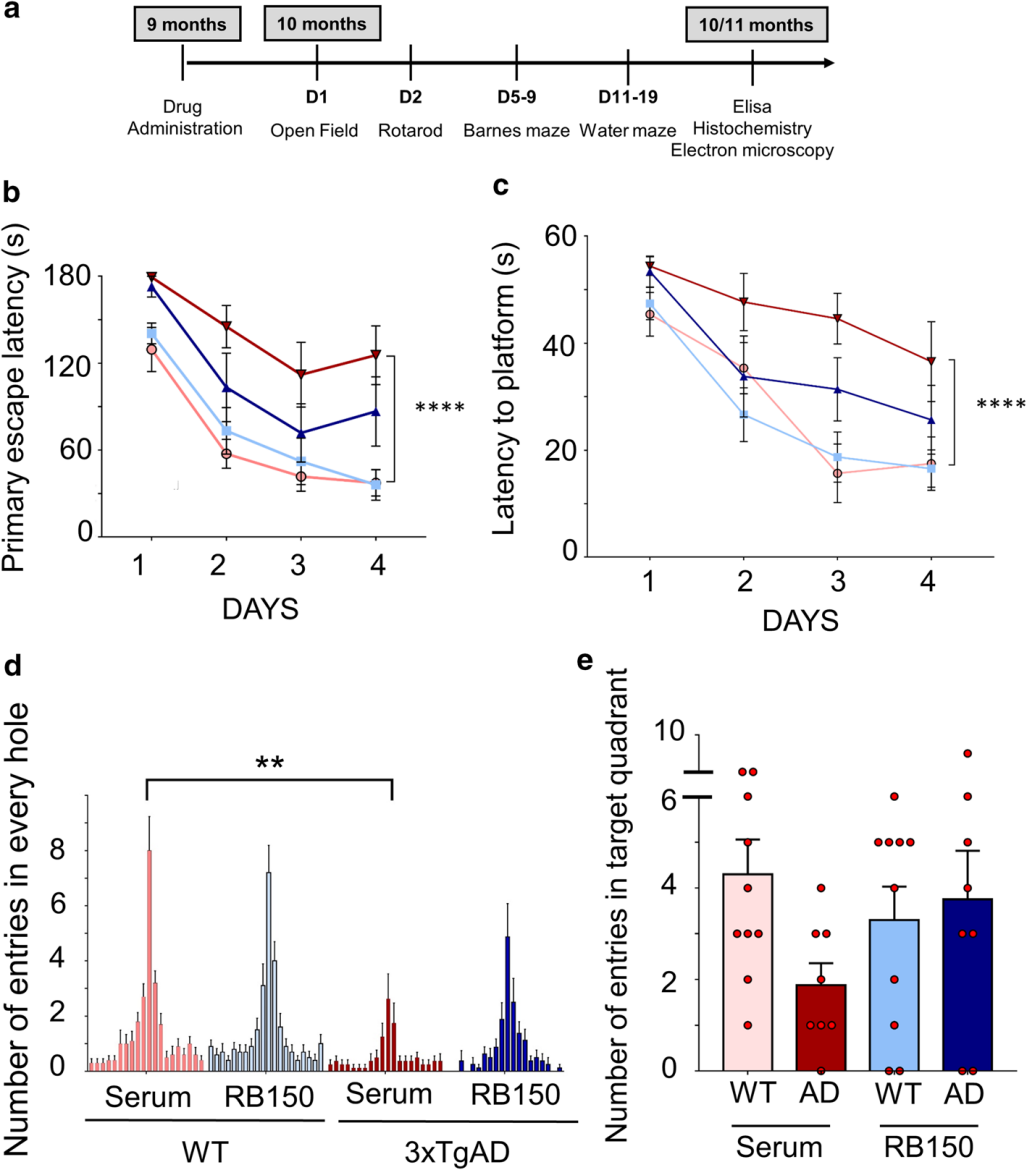
Pharmacological inhibition of APA restores learning and improves memory in 3xTgAD mice model. **a** Time schedule of behavioral analyses in mice treated with either physiological serum or RB150. **b, c** graphs represent the primary escape latency to find the target hole in the Barnes maze (**b**) and the latency to reach the platform in the Morris Water Maze (**c**) for WT and 3xTgAD mice not treated (light red and dark red, respectively) or RB150-treated (light blue and dark blue, respectively). **d, e** show the number of entries in every hole of the Barnes maze (**d)** and the number of entries in target quadrant of the MWM (**e**). Statistical analyses were performed using Anova test (WT serum: *n* = 10; WT RB150: *n* = 10; 3xTgAD serum: *n* = 8; 3xTgAD RB150: *n* = 8). Statistical analyses were performed using 2-way ANOVA test (genotype difference between 3xTgAD and WT: ****p < 0.0001 for WMW and Barnes Maze and for the number of entries in target hole: **p < 0.001)

To strengthen our data on the influence of APA gene reduction in vivo, we aimed at assessing the contribution of APA on learning deficits via pharmacological blockade of APA with RB150 in 3xTg-AD mice. After chronic treatment (daily intraperitoneal injection during 6 weeks), we monitored the behavioral paradigms identical to those described above for in vivo shAPA experiments (see time frame in Fig. 8a). As a whole, RB150 and shRNA outcomes were closely similar. Thus, RB150: (i) reduced the primary escape latency at day 5 (Fig. 8b); (ii) reduced the latency to the platform (Fig. 8c); (iii) lowered the randomness of entries in the adequate hole (Fig. 8d) and (iv) abolished the lowering in the number of entries in the target quadrant observed in 3xTg-AD mice (Fig. 8e) without affecting motricity (Suppl. Figure 9a, online resource), anxiety (Suppl. Figure 8d, online resource) and vision (Suppl. Figure 9c, online resource). Overall, our data indicate that both pharmacological reduction and gene reduction of APA partly or fully alleviate learning and spatial memory defects observed in the 3xTg-AD mice model.

### APA activity is increased early and transiently in brains of sporadic AD patients

**Fig. 9:**
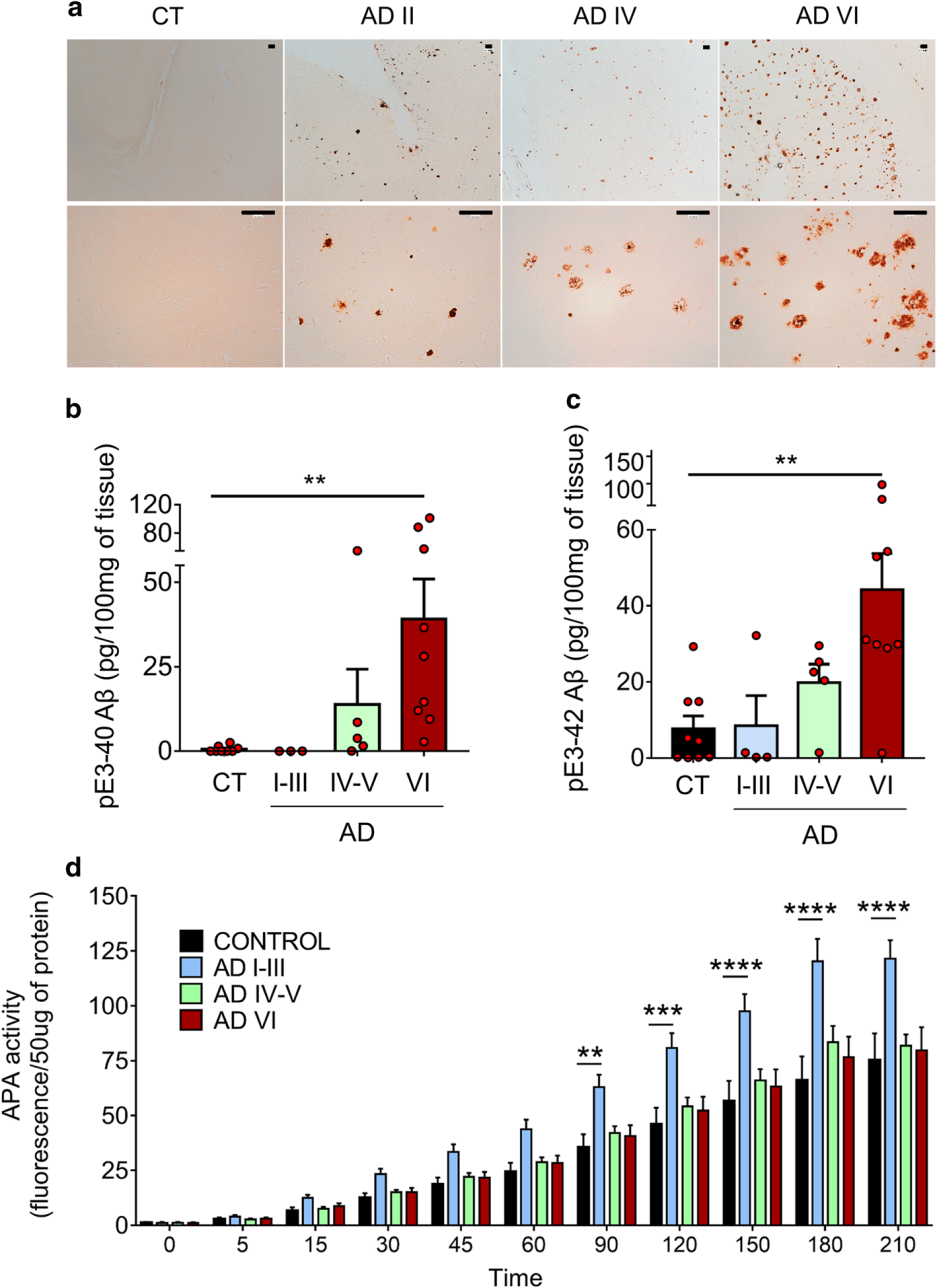
APA enzymatic activity and pE3-xAβ expression in human brain samples. **a** Immunohistochemical analysis of pE3-xAβ-positive plaques in hippocampi of control (CT) and AD-affected brains at indicated NFT Braak stages (Scale bar, 100 µm).**b**, **c** pE3-40Aβ (**b**) and pE3-42Aβ (**c**) peptides were measured in soluble fractions prepared as described in Methods by ELISA (controls: *n* = 10; AD I-III: *n* = 4; AD IV-V, *n* = 5; AD VI: *n* = 9; statistical analysis: Kruskal–Wallis, ** *p* < 0.005). **d** APA enzymatic activity was measured by fluorimetry in AD-affected samples at the indicated Braak stages (controls: *n* = 10; AD I–III: *n* = 9; AD IV-V, *n* = 10; AD VI: *n* = 14; using two-way Anova with Dunnett’s multiple comparisons post-test). **p < 0.005, ***p < 0.001; ****p < 0.0001

It has been reported that various N-terminally truncated species, and more particularly pE3-42Aβ, could be recovered in abundance in brains and cerebrospinal fluid of both sporadic and familial AD patients [19, 50]. As we demonstrate here that APA catalyzes the removal of the first aspartyl residue of Aβ, and thus trigger the first catalytic step of N-terminal truncation subsequently leading to pE3-42Aβ, we postulated that AD-related accumulation of pE3-42Aβ could be accounted for by an increased activity of APA. Thus, we examined the status of pE3-42Aβ-positive plaques, quantified pE3-42Aβ content, and monitored RB150-sensitive APA activity and expression in the temporal cortex of sporadic AD patients at various NFT Braak stages (Suppl. Table 1, online resource). We show that, as previously reported [21], AD-affected brains exhibit a high density of pE3-42Aβ-positive plaques that appeared at stage II and increased significantly according to Braak stage while control brains reveal little if any pE3-42Aβ-like immunoreactivity (Fig. 9a). Accordingly, both pE3-40Aβ (Fig. 9b) and pE3-42Aβ (Fig. 9c) levels observed in insoluble fractions of hippocampi homogenates increased with pathological stage. Of utmost interest, kinetic analyses demonstrated that RB150-sensitive APA activity was higher at early AD stage (I-III) than in control samples and then returned to control values (Fig. 9d), a phenotype confirmed by immunohistochemical analysis (Suppl. Figure 10, online resource). This transient augmentation of APA appeared concomitant to the occurrence of pE3-42Aβ-containing plaques and biochemical detection of pE3-42Aβ. It is noteworthy that, at these stages, Thal index indicates that little if any diffuse or dense-core plaques had been observed in our samples. Thus, these observations are consistent with the hypothesis of first, an early APA-linked production of pE3-42Aβ that would serve as seed to propagate anatomical lesions and second, a pE3-42Aβ accumulation without need for a sustained increase in APA activity.

## Discussion

Alzheimer’s disease-affected brains are invaded by senile plaques and neurofibrillary tangles, the main components of which consist in a set of hydrophobic peptides (Aβ peptides) that accumulate extracellularly and an hyperphosphorylated form of a microtubule-associated protein named Tau that occurs intracellularly in neurons [17, 49]. Senile plaques are preceded by diffuse plaques. The amorphous or fibrous nature of these pre-amyloid lesions is still discussed [16]. However, histochemical, immunological and biochemical analyses consistently established the occurrence of pE3-40/42Aβ as the main component of compact plaques [53] but also diffuse [31] and vascular amyloid deposits [37]. Thus, pE3-40/42Aβ is an early marker of AD pathology and, hence, could well contribute significantly to its setting and/or progression.

These observations governed the search for the protease responsible for the cyclization of the glutamate residue at position 3 of the N-terminal moiety of Aβ. Concordant pharmacological and genetic evidences indicated that the enzyme responsible for pE3-X Aβ cyclization could be ascribed to glutaminyl cyclase (QC) [15, 54, 55]. Thus, QC depletion [34] or pharmacological blockade [55] both rescue behavioral defects in transgenic AD animals. Supporting the importance of QC in AD, it was established that QC protein and mRNA expressions colocalize with pE3-40/42Aβ in AD-affected brain areas and correlates better than Aβ with cognitive alterations [44]. This set of data strongly supported the view that QC-mediated cyclisation could be a key catalytic event contributing to the neurodegenerative process taking place in AD.

Prior to cyclization yielding pE3-40/42Aβ, Aβ must undergo N-terminal truncation that removes its first aspartyl residue. Preventing this rate-limiting catalysis should be seen as a potentially protective action. The acidic nature of aspartyl and the fact that it occurs as a free residue suggested that this cleavage could be triggered by an exopeptidase with high affinity for acidic amino acids. APA fulfils these criteria [9]. Indeed, we previously established that APA inhibitors increased the recovery of full-length Aβ in various cell lines and that this protected cells from staurosporine-induced apoptosis [57]. However, the direct cleavage of Aβ by APA, its potential influence on various AD-related stigmata and its relevance in in vivo models remained to be established.

Our study brings four lines of independent evidences showing the role of APA in pE3-Aβ40/42 generation and the benefits of the reduction of its activity. First, mass-spectroscopy analysis established the conversion of Aβ1-40 into Aβ2-40. Second, pharmacological blockade of APA restored a mature dendrites morphology that is altered in APPswe-infected hippocampal organotypic slices. Third, APA inhibitors and shRNA both drastically reduced Aβ1-42- and pE3-40/42Aβ-positive plaques. Fourth, pharmacological and genetic reductions of APA activity alleviate learning and memory defects displayed by 3xTg-AD mice. It is interesting to note that, in agreement with our observations, Clark and Colleagues reported on a coincidence of synaptic plasticity alterations and spatial working memory in 3xTg-AD mice [14].

Lysosomal/endosomal perturbations [42] have been reported in AD-affected brains. We have shown that lysosomal defects occur very early in 3xTg-AD mice, at a stage where Aβ content and plaques are not detectable. We and others showed in various in vitro and mice AD models that these alterations could be ascribed to the β-secretase-derived APP C-terminal fragment (C99), rescued by β-secretase inhibitors and exacerbated by γ-secretase blockers [11, 39, 40]. APA reduction did not influence lysosomal perturbation observed in 3xTg-AD mice (data not shown). Thus, the absence of effect of APA modulation concomitant to the lack of pE3-Aβ confirms that very early deficits observed in AD could be independent of Aβ and therefore of its catabolite pE3-Aβ.

Learning and memory defects that occur later in 3xTg-AD mice were partly alleviated by APA pharmacological blockade and genetic down-regulation. This agrees perfectly with our previous conclusions brought by the comparison of 3xTg-AD and 2xTg-AD mice. Thus, both mice accumulate C99 similarly but only 3xTg-AD show plaques and Aβ-accumulation at late stage [5]. Interestingly, both mouse models exhibit early similar apathy-like phenotype and LTP alterations while older 3xTg-AD mice show higher memory deficits than 2xTg-AD mice [5]. This indicated that besides C99, Aβ or an Aβ-related catabolite could contribute to late stage memory defects. Our study strongly suggests that pE3-Aβ and APA-mediated catalytic initiation of Aβ truncation could well account for the increment of memory and learning deficits occurring in 3xTg-AD mice. The fact that APA pharmacological and genetic modulations did not fully rescue some of the behavioral defects could be due to the fact that C99 itself contributes to these deficits. Alternatively, one cannot exclude the possibility that additional exopeptidases contributing to the N-terminal truncation of Aβ could account for memory defects.

Another interesting aspect of our study is the observation that partial reduction of APA was sufficient to drastically reduce both Aβ1-42- and pE3-42Aβ-positive plaques and expressions in insoluble fractions thought to reflect an aggregated state of peptides. This agrees well with previous studies showing that pE3-42Aβ aggregates early in AD transgenic models [43] and display a prion-like behavior [45]. Further, it was documented that very low amounts of pE3-42Aβ could serve as a seed of Aβ42 aggregation and deposition [62] and that small quantities of pE3-42Aβ are sufficient to trigger selective hippocampal neurodegeneration in transgenic mice [1]. Thus, partial reduction of pE3-42Aβ by APA modulation could likely explain both the lowering of Aβ42-positive plaques and content and improvement of pE3-42Aβ-mediated behavioral alterations occurring concomitantly at same ages.

If APA is assumed to contribute to AD, its expression may be increased in affected brains as has been previously shown for several endo- and exo-peptidases [28]. Our study shows that pE3-42Aβ-positive plaques occur at Braak stage II in sporadic AD-affected brains, when pE3-42Aβ protein begins to be detectable. Interestingly, at this stage, APA activity and expression were higher in AD than in control brains. This increase appeared to be transient, suggesting that enhanced APA expression/activity could initiate a pathogenic process reflected by pE3-42Aβ-positive plaques and increased recovery. Of interest, this production is likely necessary for Aβ seeding and subsequent Aβ-positive plaques formation since our cohort indicates (according to Thal stage), that Aβ-positive plaques detection appear after APA increase and pE3-2Aβ-positive plaques.

Overall, our study reports for the first-time novel insights on the involvement of APA in the first step of Aβ catalysis ultimately leading to pE3-42Aβ in integrated models as well as in vivo. This production could account for the cognitive alterations generally ascribed to full-length Aβ. It should be emphasized that APA does not per se produce pE3-Aβ but is the limiting enzyme yielding 2–40/42 Aβ peptides. The subsequent step, i.e., the release of the Ala2 residue could be triggered by an aminopeptidase M-like activity, which displays high affinity for aliphatic residues. This APM does not release the Asp 1 residue as shown by the lack of effect of its inhibitor on the cellular recovery of full-length Aβ [57]. Another possibility would be that besides APA-mediated cleavage, a direct release of the N-terminal dipeptide Asp-Ala would occur. Our preliminary data indicate that dipeptidyl peptidase IV indeed could trigger this cleavage. Thus, we established that APA and DPPIV act independently and concomitantly since the effects of their specific inhibitors on full-length Aβ recovery in cells are additive.

It should be noted that Aβ itself derives from the proteolysis of its precursor C99 that harbors many characteristics of an etiological trigger [11, 39]. It remains that interfering with APA could be seen as a mean to alleviate, at least partly, some of the late AD-related mnesic deficits. In this context, it should be noted that RB150 crosses the blood–brain barrier after its peripheral administration, inhibits brain APA and has been successfully used to circumscribe hypertension in animals [4, 22]. Thus, we propose that the potential of APA inhibitors in AD should be examined.

## Supplementary Information

Below is the link to the electronic supplementary material.

Supplementary file1 (PDF 8667 KB)
