# Supplementary material for: Aminopeptidase A contributes to biochemical, anatomical and cognitive defects in Alzheimer’s disease (AD) mouse model and is increased at early stage in sporadic AD brain
Source: Acta Neuropathol. 2021 Apr 21;141(6):823–39. doi: 10.1007/s00401-021-02308-0 (PMC8113186; doi:10.1007/s00401-021-02308-0)
Supplement: Supplementary file 1 — Supplementary file1 (PDF 8667 KB) [file 401_2021_2308_MOESM1_ESM.pdf]

Supplementary Figures and legends:

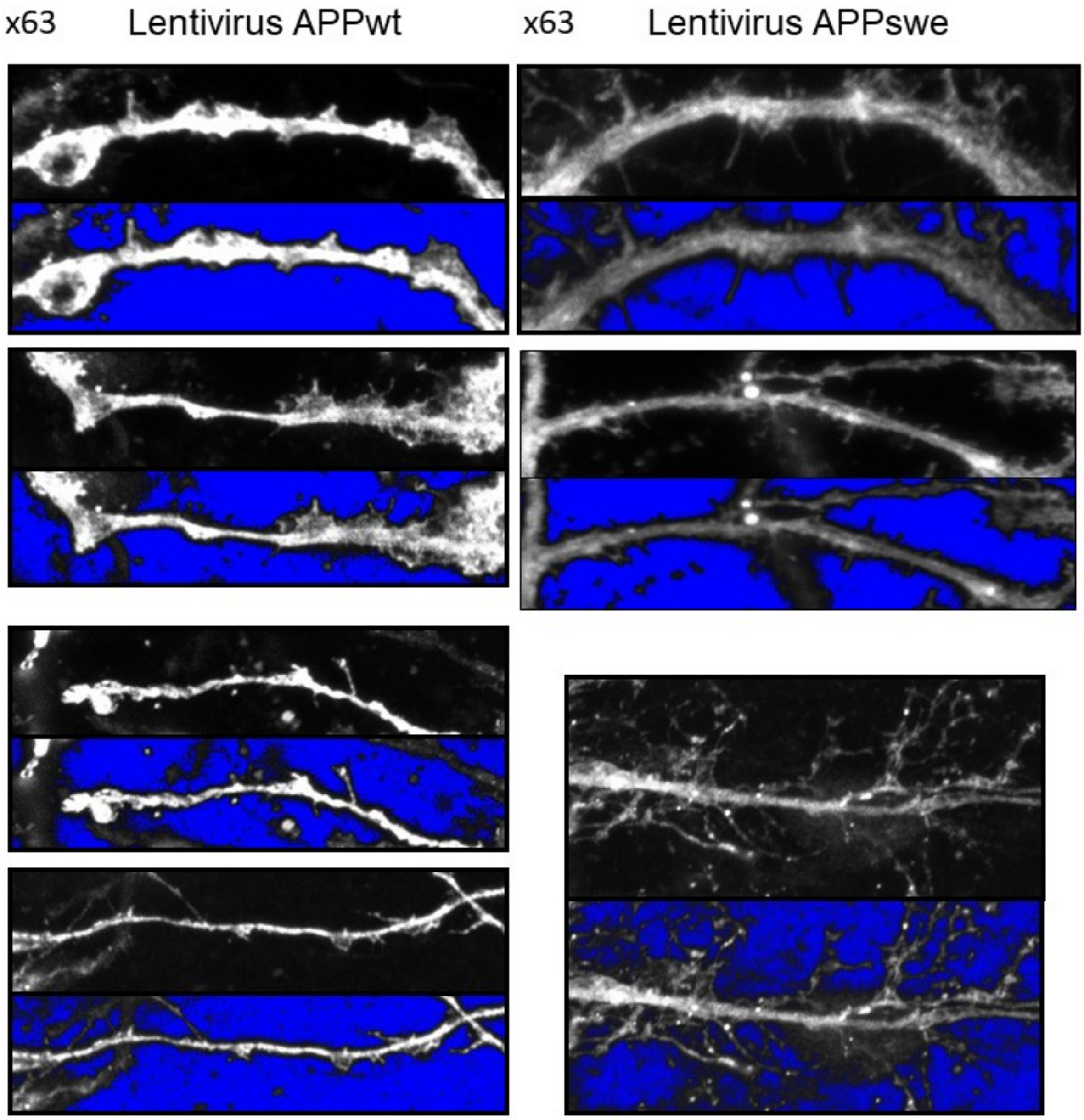

Supplementary Figure 1: Dendrites morphology in organotypic slices infected with lentiviruses expressing either APPwt or APPswe and analyzed as described in Figures 2 and 3.

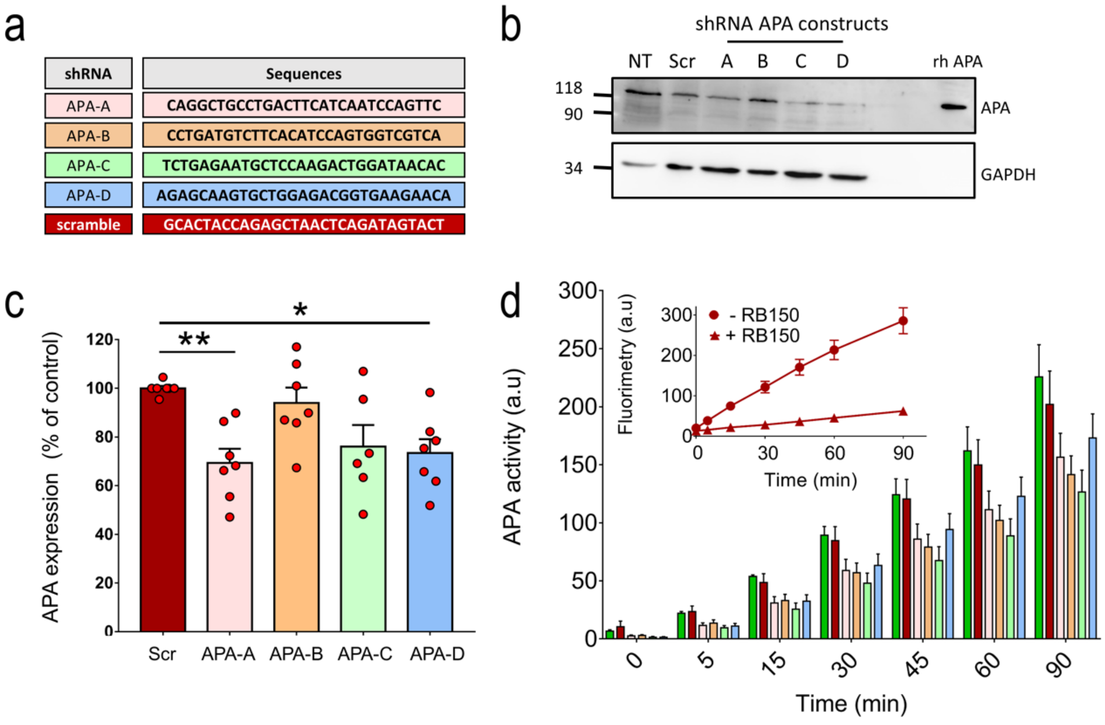

Supplementary Figure 2: a. shScr and shAPA sequences.**b,c.** APA expression (**b**) and quantitation (**c**) in N2a cells transfected with the indicated shRNA constructs. GAPDH immunoreactivity and recombinant APA (rAPA) (b) served as loading and positive controls, respectively. Values in c are expressed as percent of control (shScr-infected cells) and are means +/- SEM of: shRNA scramble: n=6; shRNA APA-A: n=7; shRNA APA-B: n=7; shRNA APA-C: n=6; shRNA APA-D: n=7. Statistical analyses were performed with one-way Anova with Dunnett's multiple comparisons post-test, *p<0.05; **<0.01). **d.** APA enzymatic activities in the above-described transfected cells as well as in naïve cells (dark green). (shScr: n=10; shAPA-A: n=14; shAPA-B: n=9; shAPA-C: n=9; shAPA-D: n=8; naïve cells: n=3). Inset in D illustrates total and RB150-resistant APA activity in shScr-infected cells

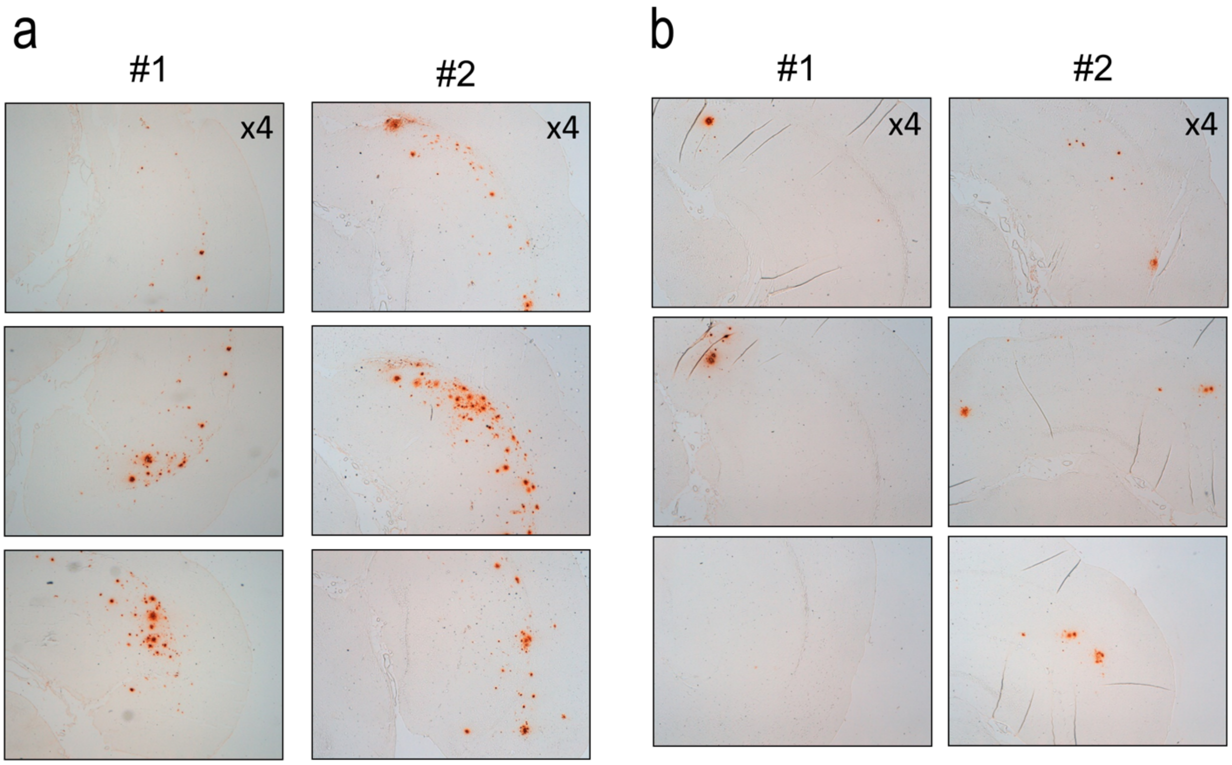

Supplementary Figure 3: Immunohistochemical analysis of Aβ42 using α-Aβ42 antibody on 12-month-old 3xTgAD transgenic mice injected with shScr (**a**) or shAPA (**b**). #1 et #2 are arbitrary numbers identifying distinct mice analyses.

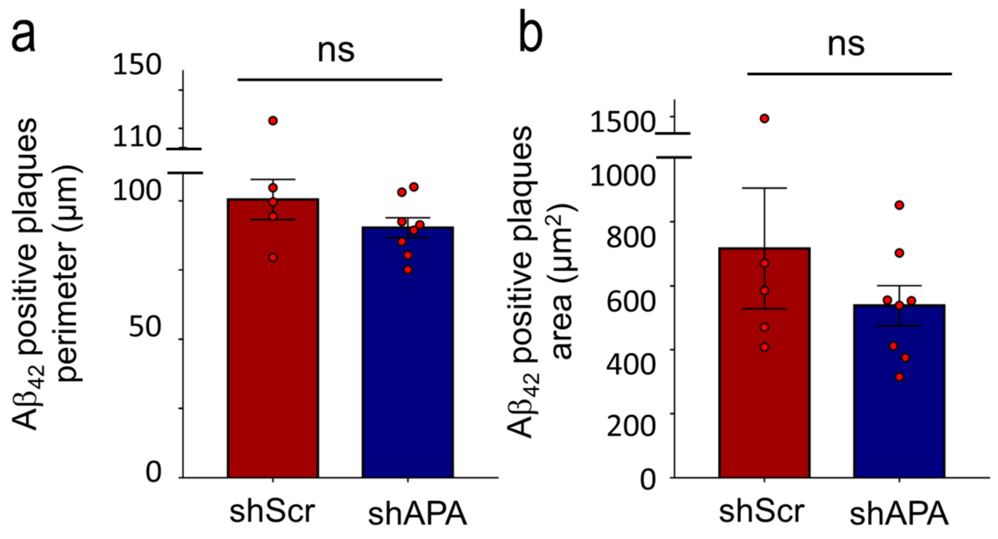

Supplementary Figure 4: Perimeter (a) and area (b) of Aβ42 positive in 12-month-old 3xTgAD transgenic mice previously injected with a shScr or shAPA. Data are represented as means +/- SEM of 5 (shScr) or 8 (shAPA), (12 pictures per mouse). Statistical analysis was performed using Mann-Whitney test. ns, non-statistically significant.

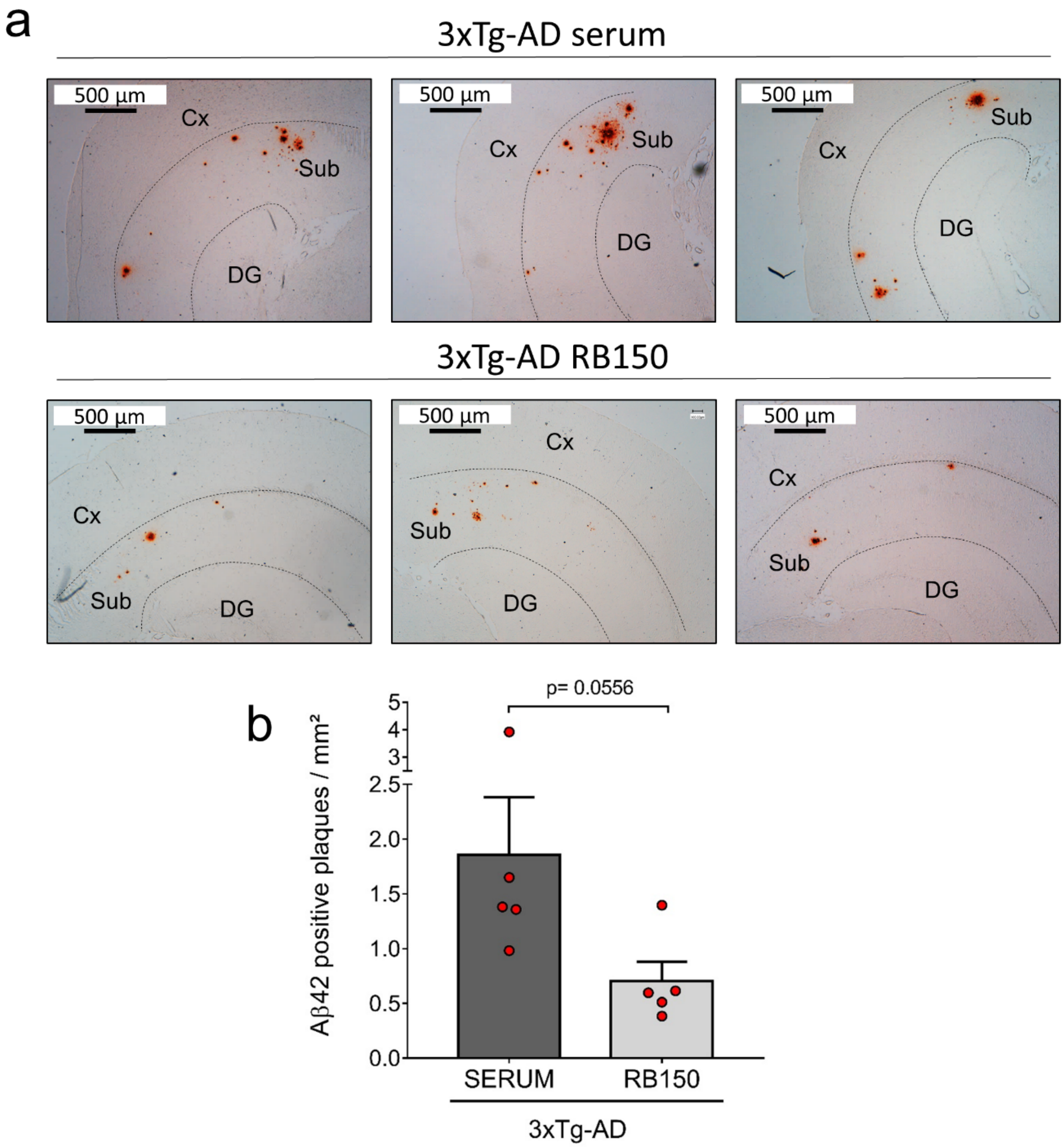

Supplementary Figure 5: a. Immunohistochemical analysis of Aβ42-like immunoreactivity in eleven-month-old three independent 3xTgAD transgenic mice after serum or RB150 treatment for six weeks. Magnification is x 4 . **b.** Graph represents the number of Aβ1-42-positive plaques per mm^2. Data represent means +/- SEM of 5 mice per condition (4 slices per mouse). Statistical analysis was performed using Mann-Whitney test.

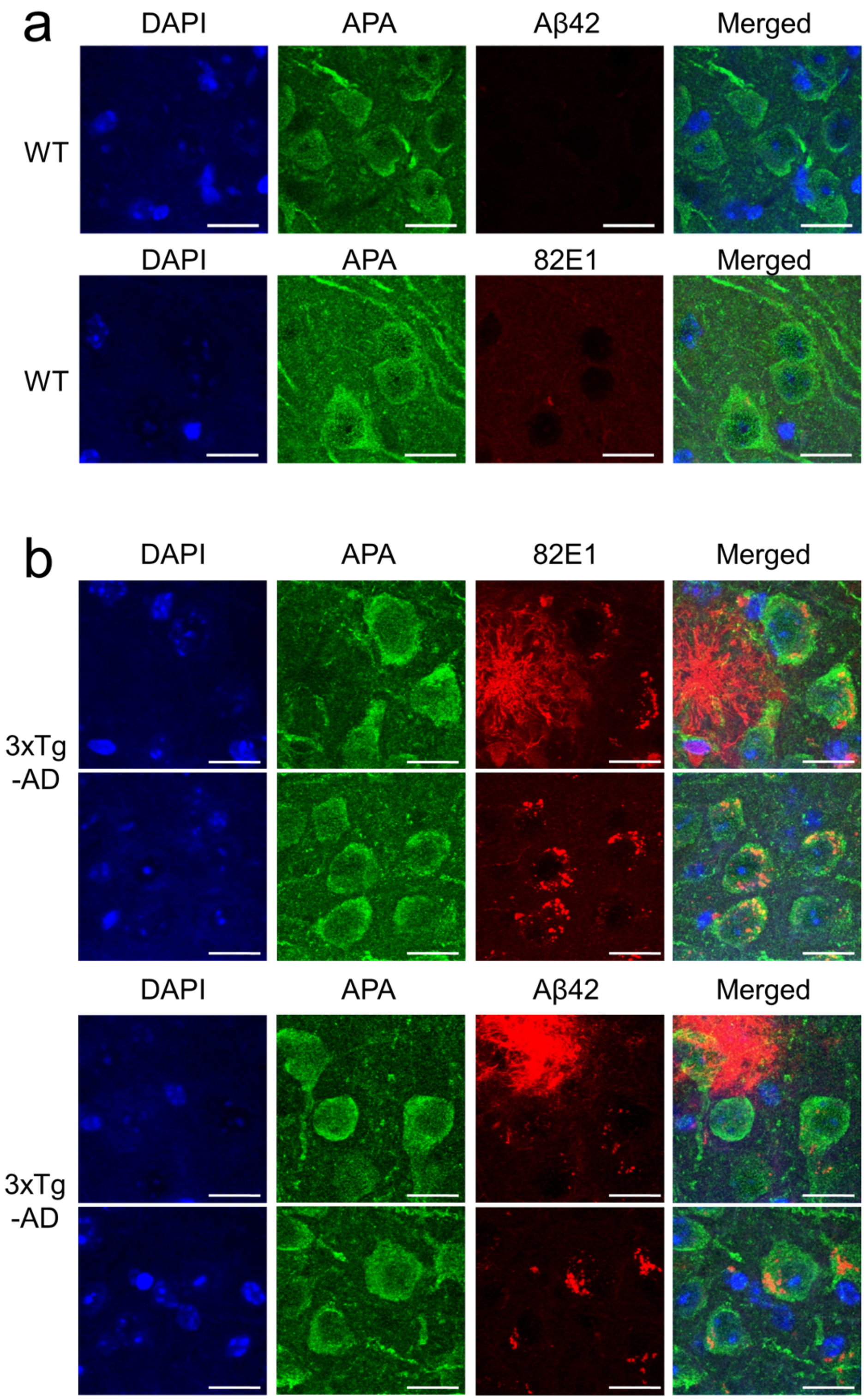

Supplementary Figure 6: 12-month-old WT (a) and 3xTgAD (b) mice infected with shScr were assessed for APA and Aβ42 co-localisation in the subiculum using Aβ-directed specific antibodies recognizing either the N-terminal (82E1) or C-terminal (Aβ42) Aβ42 moieties. Note the absence of endogenous mice Aβ-like immunoreactivity in shScr-infected WT mice (due to the use of human-specific antibodies). Nuclei were stained with Dapi. Bars correspond to 20μm

a

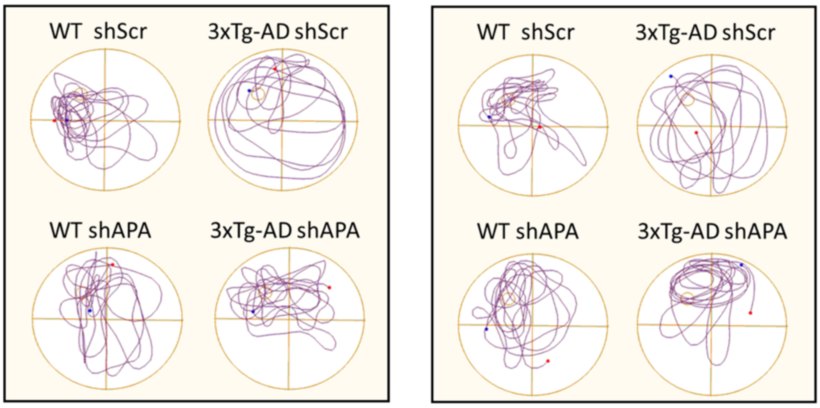

b

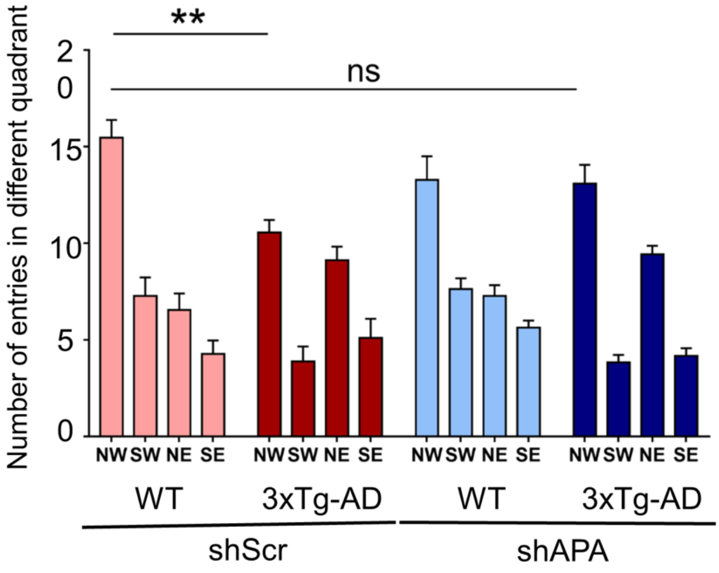

Supplementary Figure 7: Illustrations of swimming indicated mice trajectories during the probe test (**a**) and quantification of the number of entries in different quadrants of MWM (**b**). (NW: North West, SW: South West, NE: North East, SE: South East). Statistical analyses were performed using InVivoStat test (**p<0.005).

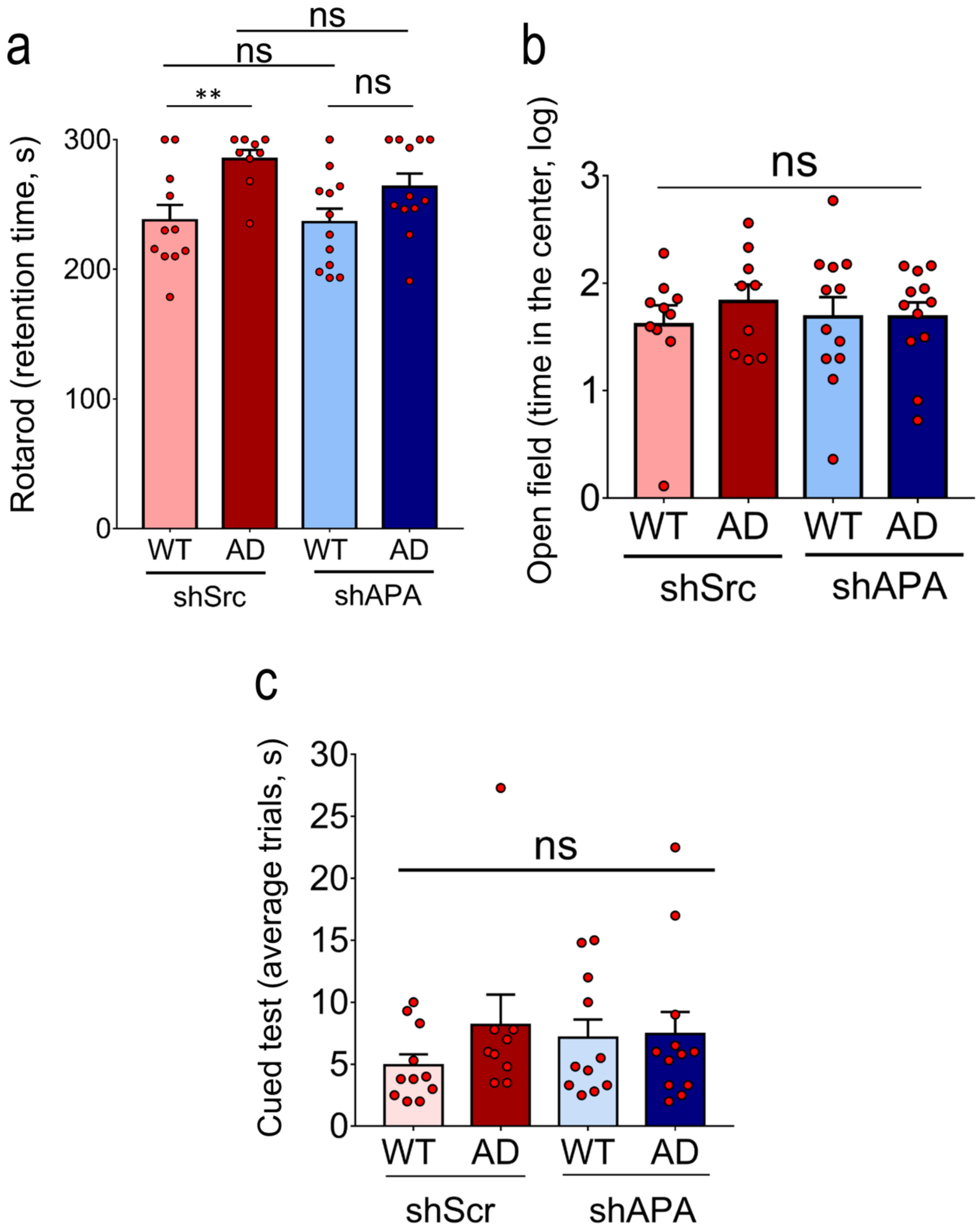

Supplementary Figure 8: 12-month-old WT and 3xTgAD (AD) mice infected with shScr or shAPA were assessed in Rotarod (**a**), open-field (**b**) and Cued test (**c**) as described in methods. Statistical analyses were performed using Anova test (WT shScr, n=11; WT shAPA, n=12; 3xTgAD shScr, n=9 and 3xTgAD shAPA, n=12). **p<0.01.

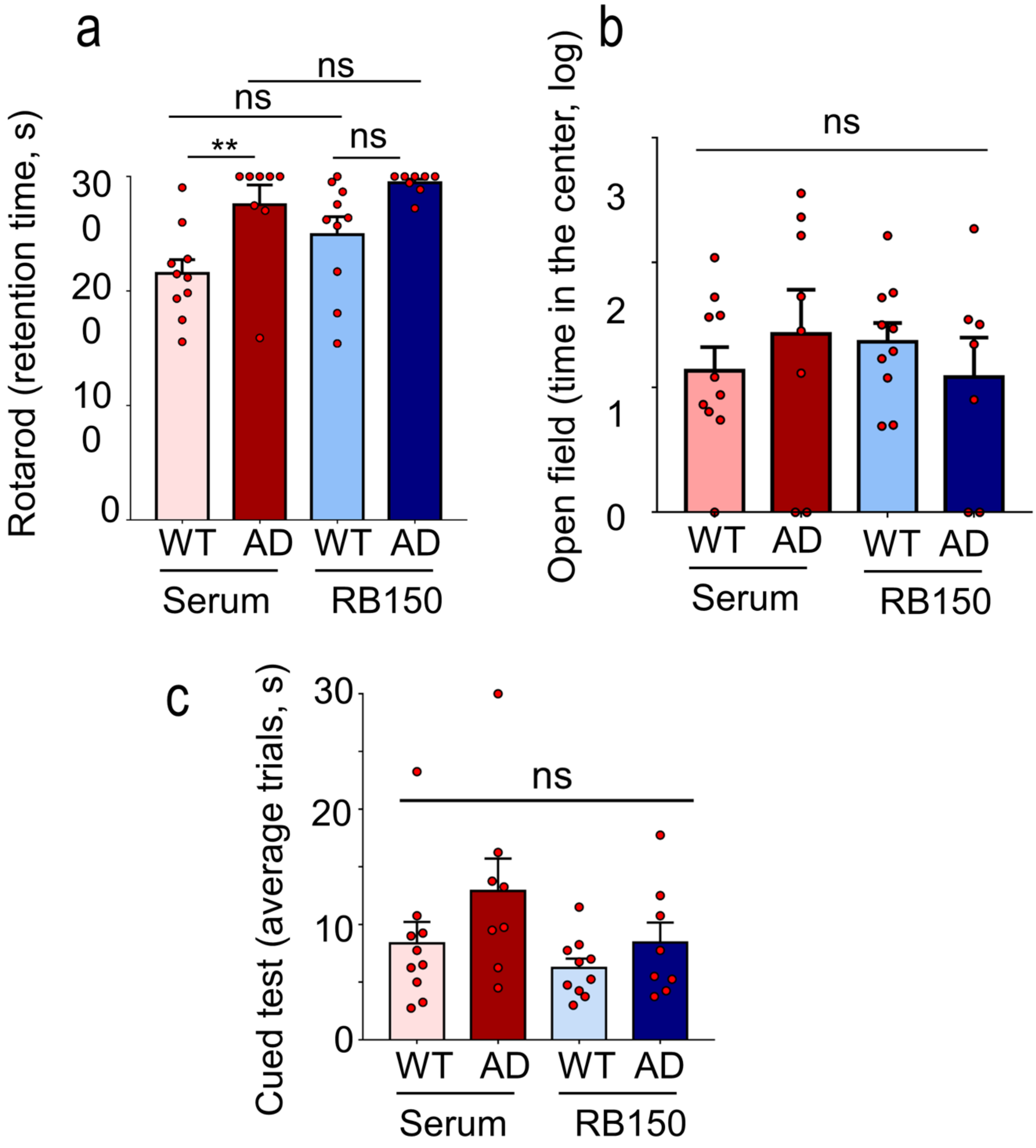

Supplementary Figure 9: 10-month-old WT and 3xTgAD (AD) mice chronically treated without (serum) or with RB150 as described in Methods were assessed in Rotarod (**a**), open-field (**b**) and Cued test (**c**) as described in methods. Statistical analyses were performed using Anova test (WT serum, n=10; WT RB150, n=10; 3xTgAD serum, n=8; 3xTgAD RB150, n=8). **p<0.01.

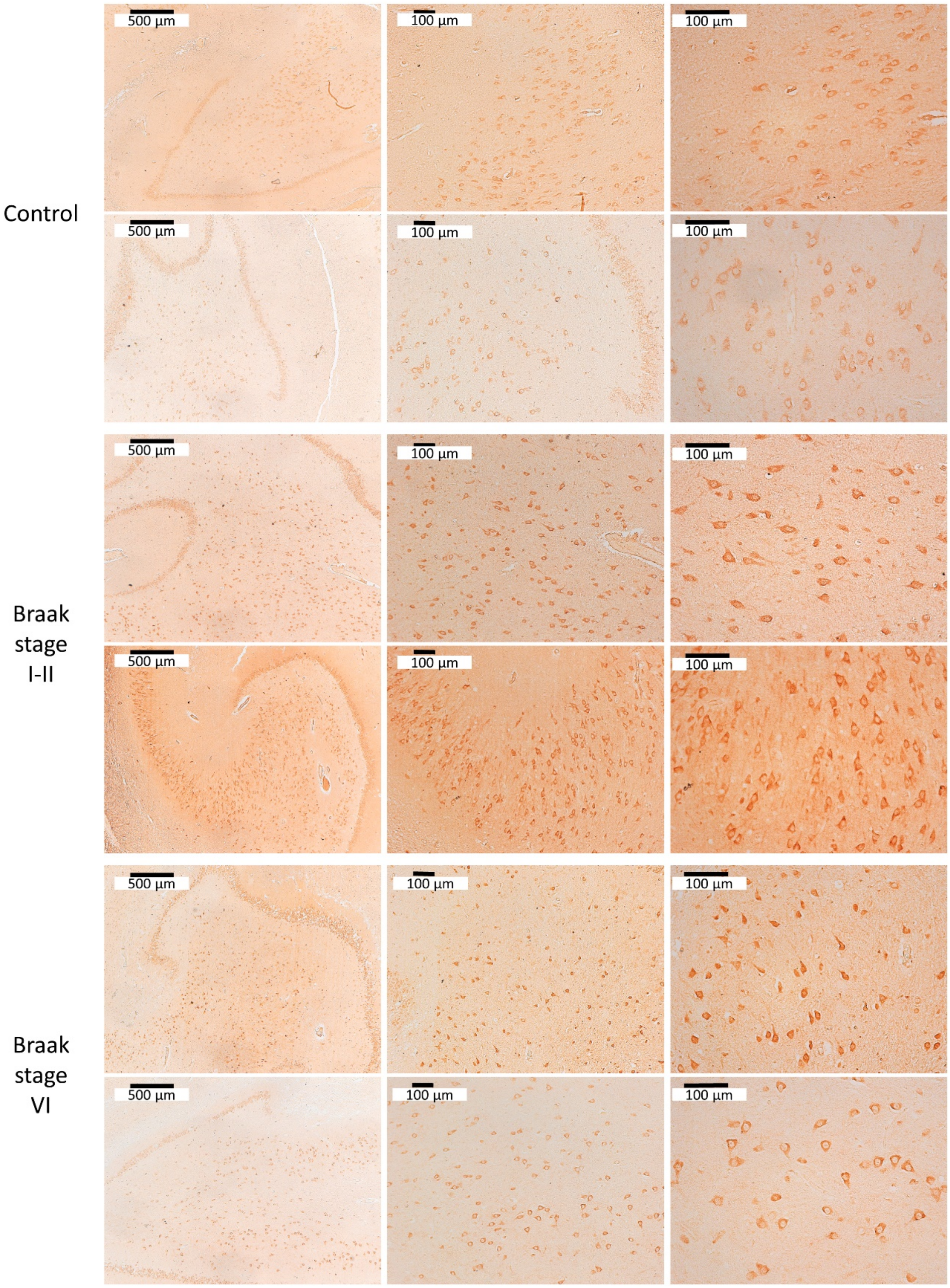

Supplementary Figure 10: Immunohistochemical analysis of APA expression in hippocampi of controls and AD-affected brains at indicated NFT Braak stages was performed as described in Methods. Magnifications are x 4 (left panels), x 10 (middle panels) and x 20 (right panels).

	Age (years)	Gender	PMD (h)	NFT Braak stage#	Thal Aβ phase	Amyloid angiopathy
**Brain samples used in enzymatic activities and Elisas analyses**						
Control	71	Male	26	-	NA	NA
Control	84	Male	32	-	NA	NA
Control	71	Female	15	-	3	2
Control	60	Female	28	-	0	0
Control	52	Female	29		0	0
Control	61	Male	20	-	3	2
ALS	62	Female	44	-	0	0
ALS	55	Male	21	-	1	0
ALS	62	Female	21	-	0	0
AD	68	Female	51	I-II	NA	NA
AD	68	Female	10	I-II	2	0
AD	92	Female	NA	III	0	0
AD	70	Male	31	III	0	0
AD	92	Female	21	I	0	2
AD	83	Female	21	II	0	0
AD	85	Male	10	II	2	2
AD	58	Male	5h30	III	0	0
AD	89	Female	45	II	2	2
AD	81	Female	60	IV	NA	NA
AD	80	Female	51	V	NA	NA
AD	80	Male	23	V	NA	NA
AD	71	Male	NA	V	NA	NA
AD	76	Female	28	IV	2	2
AD	92	Male	NA	IV	NA	NA
AD	65	Male	70	V	NA	NA
AD	82	Male	25	V	NA	NA
AD	85	Female	31	V	NA	NA
AD	84	Female	81	V	4	2
AD	81	Male	19	VI	NA	NA
AD	65	Female	41	VI	5	1
AD	89	Female	26	VI	4	2
AD	91	Female	34	VI	5	1
AD	55	Female	58	VI	5	1
AD	75	Female	7	VI	5	2
AD	81	Female	NA	VI	5	1
AD	82	Female	NA	VI	4	1
AD	67	Male	30	VI	5	2
AD	86	Male	31	VI	5	1
AD	69	Male	30	VI	5	2
AD	81	Male	34	VI	4	2
AD	84	Female	52	VI	5	1
AD	76	Male	27	VI	5	2
**Brain-derived slices used in pE3-42Aβ immunohistochemical analyses**						
Control	55	Male	NA	-	NA	NA
AD	81	Male	NA	II	NA	NA
AD	77	Female	NA	IV	NA	NA
AD	83	Male	NA	VI	NA	NA
**Brain-derived slices used in APA immunohistochemical analyses**						
Control	55	Male	NA	-	NA	NA
Control	62	Female	NA	-	NA	NA
AD	68	Female	NA	I-II	NA	NA
AD	61	Male	NA	I	NA	NA
AD	62	Male	NA	VI	NA	NA
AD	82	Female	NA	VI	NA	NA

Supplementary Table 1: Neuropathological findings related to human brain samples used in enzymatic activities and Elisas analyses (temporal lobe), and brain-derived slices used in immunohistochemistry analyses (T1 region of the temporal lobe). Neuropathological characterization has been done on each sample (NFT Braak stage, Thal Aβ phase and amyloid angiopathy). PMD: Post mortem delay, h: hours, NA: not available. Controls and ALS are brain samples isolated from post-mortem patients diagnosed as negative for AD pathology obtained from post-mortem patients diagnosed or not with Amyotrophic lateral sclerosis. (-) means no NFT detection.
